# Neonatal hyperoxia inhibits proliferation and survival of atrial cardiomyocytes by suppressing fatty acid synthesis

**DOI:** 10.1172/jci.insight.140785

**Published:** 2021-03-08

**Authors:** Ethan David Cohen, Min Yee, George A. Porter, Erin Ritzer, Andrew N. McDavid, Paul S. Brookes, Gloria S. Pryhuber, Michael A. O’Reilly

**Affiliations:** 1 Department of Pediatrics,; 2 Department of Biostatistics & Computational Biology, and; 3 Department of Anesthesiology, School of Medicine and Dentistry, The University of Rochester, Rochester, New York, USA.

**Keywords:** Cardiology, Cardiovascular disease, Fatty acid oxidation, Mouse models

## Abstract

Preterm birth increases the risk for pulmonary hypertension and heart failure in adulthood. Oxygen therapy can damage the immature cardiopulmonary system and may be partially responsible for the cardiovascular disease in adults born preterm. We previously showed that exposing newborn mice to hyperoxia causes pulmonary hypertension by 1 year of age that is preceded by a poorly understood loss of pulmonary vein cardiomyocyte proliferation. We now show that hyperoxia also reduces cardiomyocyte proliferation and survival in the left atrium and causes diastolic heart failure by disrupting its filling of the left ventricle. Transcriptomic profiling showed that neonatal hyperoxia permanently suppressed fatty acid synthase (*Fasn*), stearoyl-CoA desaturase 1 (*Scd1*), and other fatty acid synthesis genes in the atria of mice, the HL-1 line of mouse atrial cardiomyocytes, and left atrial tissue explanted from human infants. Suppressing *Fasn* or *Scd1* reduced HL-1 cell proliferation and increased cell death, while overexpressing these genes maintained their expansion in hyperoxia, suggesting that oxygen directly inhibits atrial cardiomyocyte proliferation and survival by repressing *Fasn* and *Scd1*. Pharmacologic interventions that restore *Fasn*, *Scd1*, and other fatty acid synthesis genes in atrial cardiomyocytes may, thus, provide a way of ameliorating the adverse effects of supplemental oxygen on preterm infants.

## Introduction

Approximately 10% of births occur before 37 weeks of gestation and are, thus, considered preterm. Children who are born preterm face an increased risk of developing airway hyperreactivity, chronic wheezing, emphysema, and other respiratory diseases as children and young adults compared with those born at term (1–5). Now that more and more severely preterm infants are reaching adulthood, it is becoming clear that these individuals are also predisposed to pulmonary vascular disease and heart failure (6, 7). Most strikingly, people who were born extremely preterm are 4.6 times more likely to develop pulmonary hypertension and 17 times as likely to suffer heart failure as young adults than people who were born at term (8). While prior studies have reported a loss of pulmonary capillaries, altered ventricular shape, and decreased aortic size in young adults born preterm (9–13), the root causes of pulmonary hypertension, heart failure, and other cardiovascular diseases in the survivors of preterm birth remain poorly understood. Recent echocardiographic imaging, MRI, and CT imaging studies indicate that preterm infants have larger left atria and lower diastolic function than term infants (14–17). These changes may be an early and direct response to oxygen exposure because diastolic heart failure has been seen in preterm infants on supplemental oxygen therapies (18).

We and other investigators have been using rodents to understand how early-life exposure to high levels of oxygen (hyperoxia) causes cardiovascular disease. In our model, newborn mice are exposed to room air or hyperoxia (100% oxygen) from birth to P4 and recovered in room air. Mice exposed to neonatal hyperoxia develop pulmonary diseases like those of former preterm infants, including alveolar simplification nonatopic airway hyperreactivity (19) and reduced lung function (20). They also have persistent inflammation and develop fibrotic lung disease after influenza A infection (21, 22). Additionally, hyperoxia-exposed mice develop pathological symptoms of pulmonary hypertension that include pulmonary capillary rarefaction, right ventricular hypertrophy, and a 50% mortality by 1 year of age (23). Other investigators have similarly observed cardiovascular disease, including right ventricular hypertrophy, hypertension, capillary rarefication, and other types of vascular dysfunction, in animals exposed to neonatal hyperoxia (24–28). Together, these findings demonstrate that neonatal hyperoxia can cause pulmonary hypertension and other cardiovascular diseases in adult rodents.

We recently reported that hyperoxia inhibits the proliferation of the cardiomyocytes wrapping the pulmonary vein and prevents them from expanding to cover its distal branches within the growing lungs of neonatal mice (29). Since these cells form a contractile sleeve around the pulmonary vein and extending into the left atria, the early loss of these cells may increase the force needed to drive pulmonary circulation and, thus, contribute to the development of pulmonary venous congestion and heart failure in aged hyperoxia-exposed mice (30). Herein, we report that neonatal hyperoxia inhibits the proliferation of left atrial cardiomyocytes by suppressing genes required for the de novo synthesis of fatty acids. Failure to expand these cells results in hypoplastic left atria that lose the ability to effectively fill the left ventricle as mice age, a finding that may help explain the pathogenesis of diastolic heart failure in the survivors of preterm birth.

## Results

### Neonatal hyperoxia inhibits left atrial cardiomyocyte proliferation.

**Figure 1:**
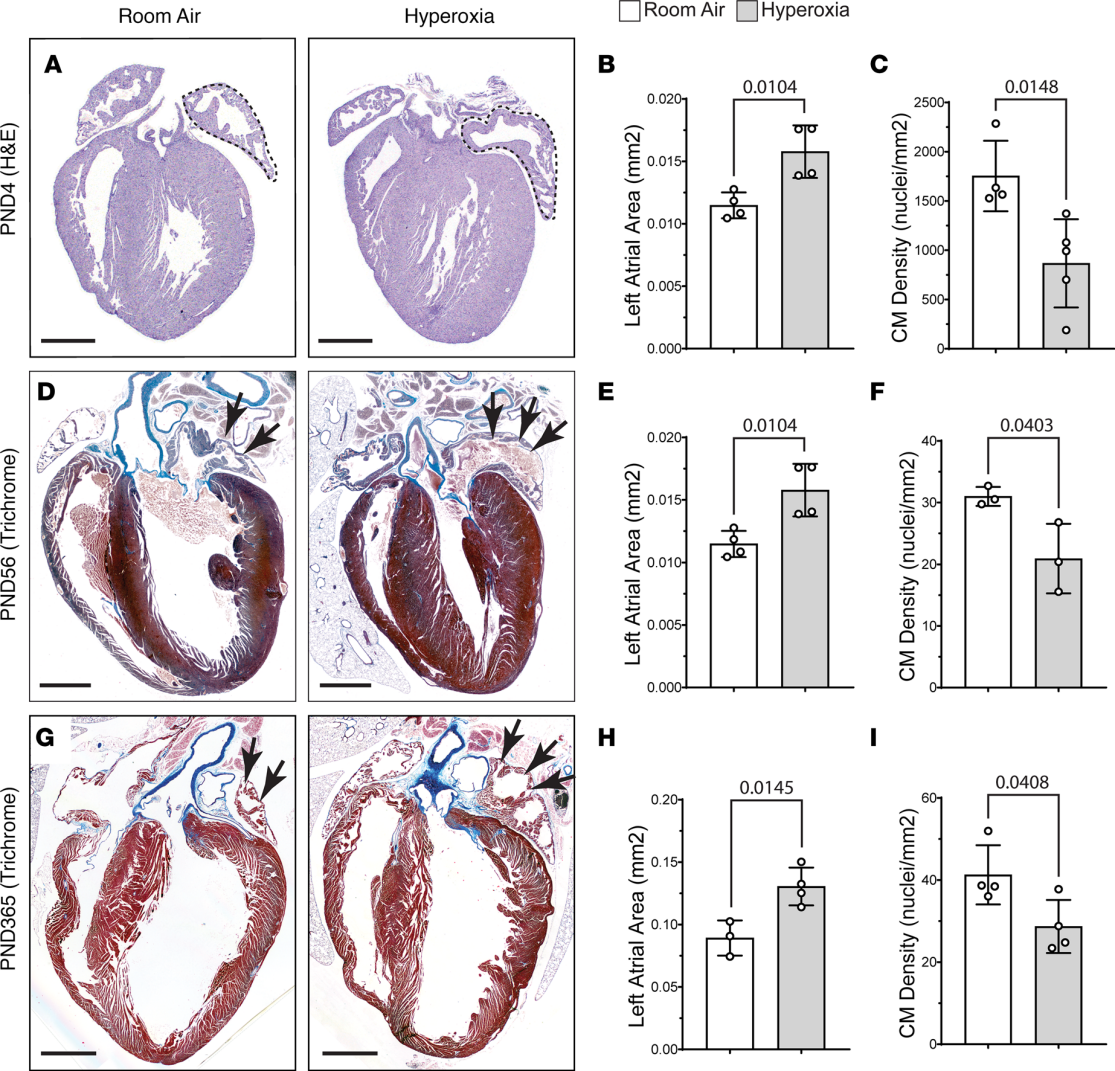
Neonatal hyperoxia enlarges the left atria of mice but reduces the density of cardiomyocyte (CM) nuclei in the left atrial myocardia. (**A**) H&E-stained sections of hearts from P4 mice that were exposed to room air (left) or hyperoxia (right). Dotted lines outline the left atria. (**B**) Mean area of the left atria in sections of room air– and hyperoxia-exposed mice on P4. *n* = 4 mice per condition. (**C**) Numbers of DAPI-stained nuclei per mm^2^ of TNNT2^+^ myocardium. Room air, *n*= 4; hyperoxia, *n* = 5 mice per condition. (**D** and **G**) Sectioned hearts of P56 (**D**) and P365 (**G**) mice exposed to room air (right) or hyperoxia (right) from P0–P4 stained with Masson’s trichrome. (**E** and **H**) Graphs show mean area of the left atria in sections of room air– and hyperoxia-exposed mice on P56 (**E**) and P365 (**F**). (**E**) *n* = 4 per condition. (**H**) Room air, *n* = 3; hyperoxia, *n* = 4. (**F** and **I**) Graphs show numbers of DAPI-stained nuclei per mm^2^ of TNNT2^+^ myocardium in room air– and hyperoxia-exposed mice on P56 (**F**) and P365 (**I**). (**F**) *n* = 3 mice per condition. (**I**) *n* = 4 mice per condition. Bars in graphs show mean values, circles show individual data points, and error bars show SDs. The *P* values between data sets are from unpaired 2-tailed *t* tests. Scale bars: 400 µm (**A**) or 100 µM (**D** and **G**).

**Figure 2:**
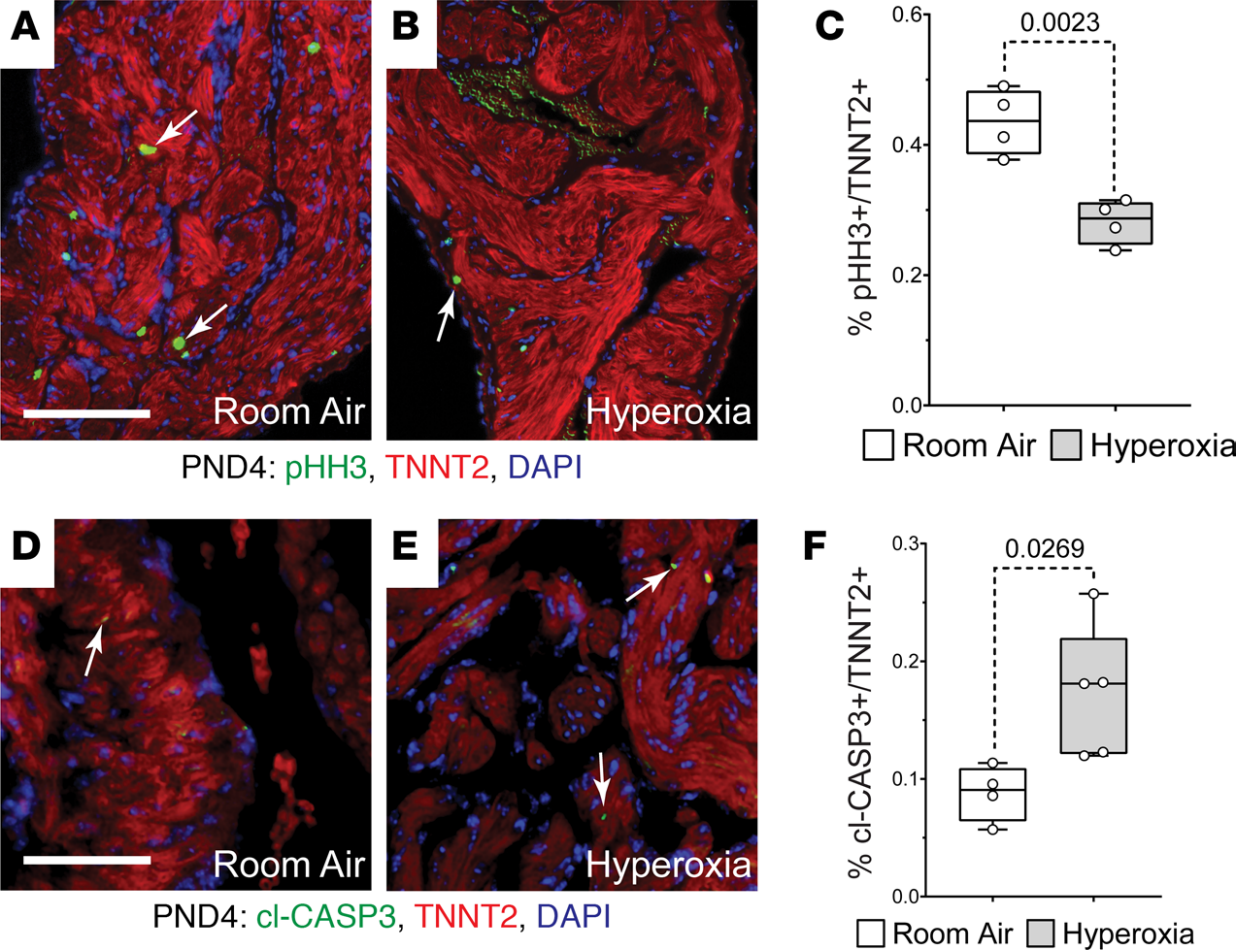
Hyperoxia reduces postnatal proliferation and survival of left atrial cardiomyocytes in neonatal mice. (**A** and **B**) Sections of left atrial appendages from P4 control (**A**) and hyperoxia-exposed (**B**) mice stained with phosphorylated Histone H3 (pHH3, green), cardiac troponin T (TNNT2, red), and DAPI (blue). Arrows point to pHH3^+^ cardiomyocytes. (**C**) Percentage of pHH3^+^ cardiomyocytes in P4 mice exposed to room air or hyperoxia. *n* = 4 mice per condition. (**D** and **E**) Sectioned left atrial appendages of P4 control (**D**) and hyperoxia-exposed (**E**) mice stained for the active, cleaved form of Caspase 3 (cl-Casp3), TNNT2, and DAPI. Arrows point to cl-Casp3^+^ cardiomyocytes. (**F**) Percentage of cl-Casp3–labeled CMs in the left atria of P4 hyperoxia-exposed and control mice. Room air, *n* = 4; hyperoxia, *n* = 5 mice per condition. Box plots show median, second quartiles, and third quartiles; whiskers indicate range, and circles represent individual data points. *P* values are results of unpaired 2-tailed *t* tests. Scale bars: 200 μm.

We previously showed that hyperoxia causes a loss of the cardiomyocytes wrapping the pulmonary vein by inhibiting their proliferation (29). Since the pulmonary vein cardiomyocytes are contiguous with the left atria, we investigated whether neonatal hyperoxia altered left atrial morphology. Counterintuitively, H&E staining revealed that the left atria of mice exposed to neonatal hyperoxia were larger than those of controls on P4 (dotted lines; Figure 1, A and B). However, costaining for the cardiomyocyte marker cardiac tropoinin T (TNNT2) and DAPI showed the number of nuclei per mm^2^ of TNNT2^+^ myocardium is reduced in the left atria of P4 hyperoxia-exposed mice relative to controls (Figure 1C). The left atria of mice exposed to neonatal hyperoxia remained larger and continued to have fewer nuclei in TNNT2^+^ regions of the left atria than controls on P56 (arrows; Figure 1, D–F) and P365 (arrows; Figure 1, G–I). Sectioned hearts of P4 hyperoxia-exposed and room air control mice were next stained for TNNT2 and the mitosis marker phosphorylated histone H3 (pHH3) or cleaved caspase 3 (cl-CASP3), which labels apoptotic cells. Fewer pHH3^+^ (arrows; Figure 2, A–C) and more cl-CASP3^+^ (arrows; Figure 2, D–F) cardiomyocytes were observed in the left atria of mice exposed to neonatal hyperoxia than controls. Together, these data suggest that neonatal hyperoxia reduced the proliferation and survival of left atrial cardiomyocytes.

### Neonatal hyperoxia causes diastolic dysfunction in adult mice.

To determine how neonatal hyperoxia affects adult heart function, echocardiography was first performed on five P56 mice that were exposed to room air or hyperoxia between P0 and P4 and an equal number of room air controls (Supplemental Table 1; supplemental material available online with this article; https://doi.org/10.1172/jci.insight.140785DS1). Hyperoxia did not affect left ventricular systolic function on P56, but the calculated mass of the left ventricular wall was lower in hyperoxia-exposed mice than controls. Since a prior study showed that hyperoxia reduced ventricular cardiomyocyte proliferation (31), the reduced density of the left ventricular wall observed by echocardiography may reflect lower densities of cardiomyocytes in the left ventricular myocardium. Doppler imaging was used to measure the maximum velocity (Vmax) of blood flowing across the mitral valve during early diastole when the left ventricle relaxes (E-peak) and late diastole when the left atrium contracts (A-peak). While hyperoxia did not affect either the E- or A-peak Vmax at this age, 3 of the 5 hyperoxia-exposed mice had E/A ratios > 2, which is above normal range in humans and mice (32–34).

**Figure 3:**
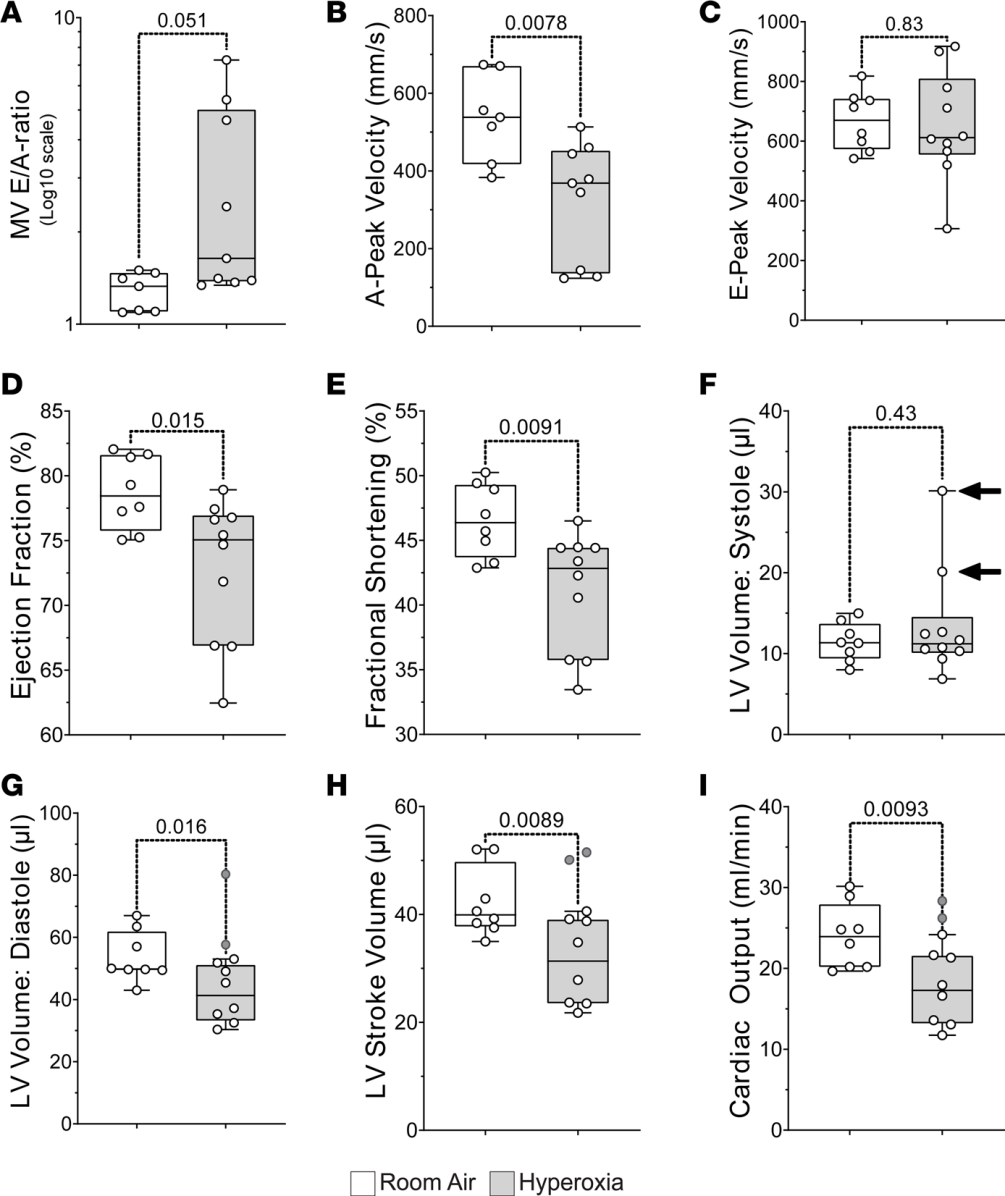
Neonatal hyperoxia causes mice to develop dilated heart failure later in life. (**A**–**C**) Graphs show mean E/A ratios (**A**), A-peak velocities (**B**), and E-peak velocities (**C**) of blood flow across the mitral valves of control and hyperoxia-exposed mice on P365. E/A ratios are plotted on a log_10_ scale. (**D** and **E**) Graphs show mean ejection fraction (**D**) and fractional shortening (**E**) of control and hyperoxia-exposed mice on P365. (**F**) Graph shows mean volume of the left ventricle during systole for PD365 control and hyperoxia-exposed mice. Arrows point to 2 hyperoxia-exposed mice with left ventricles that were drastically enlarged relative to controls and other hyperoxia-exposed mice. These 2 mice had the highest E/A ratios of all mice. (**G**–**I**) Box plots show the mean diastolic volume (**G**), stroke volume (**H**), and cardiac output (**I**) of the left ventricles in P365 hyperoxia-exposed and control mice after the 2 mice with enlarged left ventricular systolic volumes were excluded. Gray circles are values for excluded mice. Box plots show median, second quartiles, and third quartiles; whiskers show range, and circles show individual data points. *F* tests were used to determine if values for control and hyperoxia-exposed mice had equal variances. The *P* values are from unpaired 2-tailed *t* tests.

Cardiac function was next examined in 10 hyperoxia-exposed and 8 room air control mice on P365 to determine how the effects of neonatal hyperoxia progress with age (Supplemental Table 2). Four of the 10 hyperoxia-exposed mice examined at this age had E/A ratios > 2 like those observed on P56. Moreover, the mean E/A ratio was higher in hyperoxia-exposed mice than controls on P365 (Figure 3A), although the *P* value for this change was slightly above the threshold for significance (unpaired 2-tailed *t* test, *P* = 0.051). Interestingly, mean A-peak Vmax was lower in hyperoxia-exposed mice than controls at this age, while E-peak Vmax was unaffected (Figure 3, B and C). The ratio of E-peak velocity to mitral annular velocity (E/e’), an indicator of left atrial pressure in early diastole, was also unaffected (room air, –32.34 ± 8.36, *n* = 8; hyperoxia, –30.33 ± 5.92, *n* = 10; *P* = 0.56). Neonatal hyperoxia may, thus, specifically disrupt the later active phase of diastole, which is driven by atrial contraction, without affecting early diastole, which is driven by ventricular relaxation. Ejection fraction and fractional shortening were also significantly reduced in P365 hyperoxia-exposed mice relative to controls (Figure 3, D and E). Neonatal hyperoxia did not significantly affect mean systolic volume of the left ventricle, but the 2 mice with the highest E/A ratios had hearts with much higher left ventricular systolic volumes than controls that were identified as statistical outliers (arrows; Figure 3F). The ventricular dilation in the most severely affected mice may help maintain cardiac output in the setting of diastolic dysfunction. In support of this idea, the 2 hyperoxia-exposed mice with the highest E/A ratios and systolic volumes also had higher diastolic volumes, stroke volumes, and cardiac outputs than the remaining hyperoxia-exposed mice (gray circles; Figure 3, G–I). The mean diastolic volume, stroke volume, and cardiac output of the remaining mice were also reduced in the remaining mice exposed to neonatal hyperoxia relative to those exposed to room air when these 2 individuals were excluded from the analysis (white circles, box plots, and *P* values; Figure 3, G–I). Together, these data suggest that neonatal hyperoxia causes mice to develop diastolic dysfunction as adults (P56) that progresses to heart failure by middle age (P365).

### Neonatal hyperoxia inhibits the de novo fatty acid synthesis pathway in the atria of mice.

**Figure 4:**
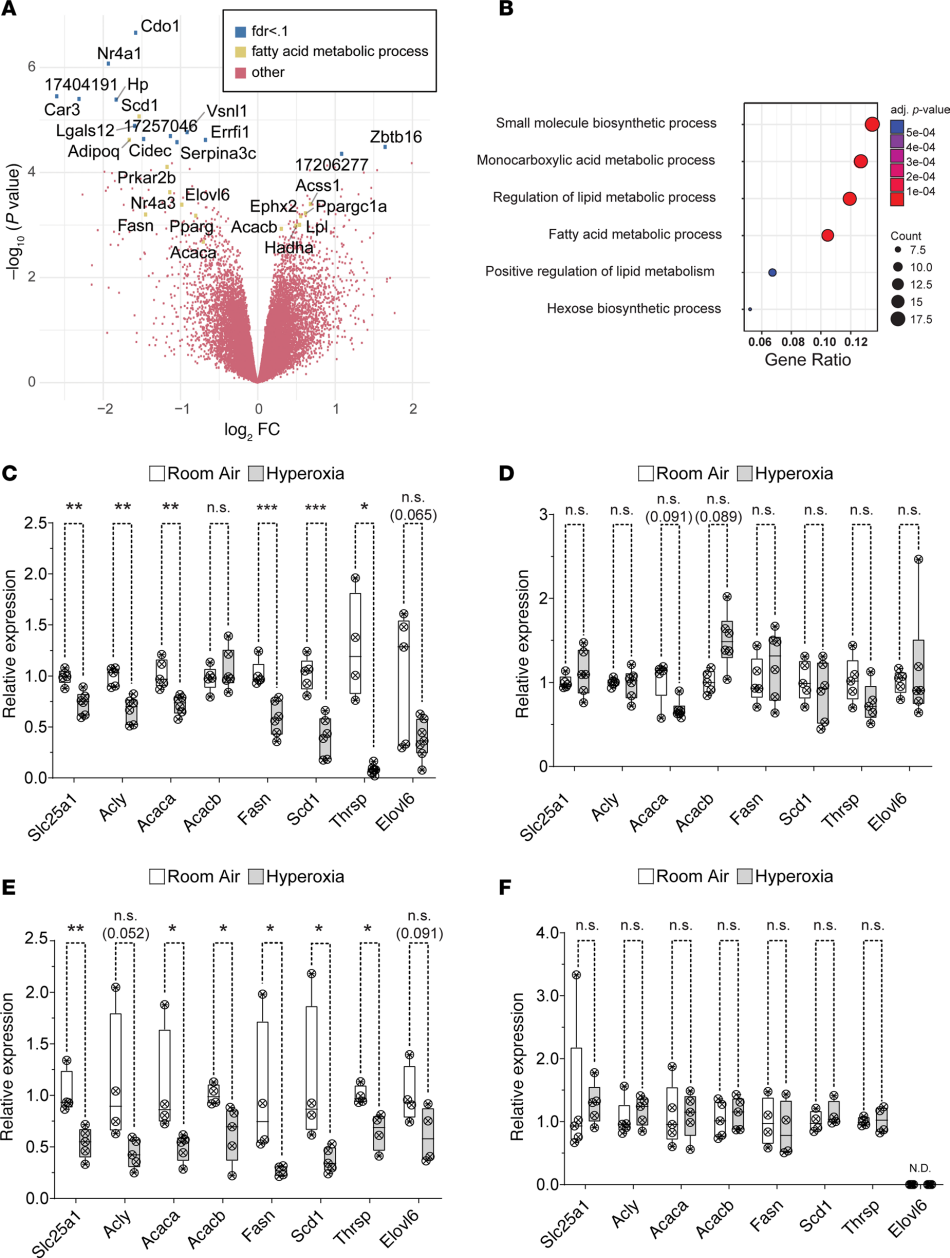
Neonatal hyperoxia suppresses genes needed for fatty acid synthesis in atria of mice. (**A**) Volcano plot of log_2_ fold changes versus log_10_ *P* values. Genes with FDR < 0.1 or annotation in the fatty acid metabolism are highlighted. Genes with FDR < 0.1 are marked by blue dots; genes involved in fatty acid metabolism are marked by green dots. (**B**) Gene ontology (GO) analysis of differentially expressed genes. (**C**–**F**) Results of qPCR for *Slc25a1, Acly, Acaca, Acacb*, *Fasn*, *Scd1*, *Thrsp*, and *Elovl6* in atria (**C** and **E**) and ventricles (**D** and **E**) of hyperoxia-exposed and control mice on P4 (**C** and **D**) and P56 (**E** and **F**). (**A** and **B**) Room air, *n* = 3; hyperoxia, *n* = 4. (**C**–**E**) Room air, *n* = 5; hyperoxia, *n* = 6. (**F**) *Slc25a1, Acly, Acaca, AcacB*: room air, *n* = 5; hyperoxia, *n* = 5; *Scd1, Fasn, Thrsp, Elovl6*: room air, *n* = 4; hyperoxia, *n* = 4. (**C**–**F**) Box plots show median, second quartiles, and third quartiles; whiskers show range. Circles show values for individual control and hyperoxia-exposed mice, respectively. (**C**–**F**) *P < 0.05, **P < 0.01, ***P < 0.005, using unpaired 2-tailed *t* tests.

An Affymetrix array was used to identify hyperoxia-induced changes in gene expression responsible for the inhibition of left atrial cardiomyocyte proliferation. RNA was isolated from the atria of 4 hyperoxia-exposed and 3 room air control mice on P4 and hybridized to the array. Out of 39,000 transcripts examined, only 158 differed by ≥ 1.5-fold with a *P* < 0.05 and FDR < 0.3 (Figure 4A). Neonatal hyperoxia increased expression of 53 genes (Supplemental Table 3) and inhibited expression of 105 genes (Supplemental Table 4). Gene ontology (GO) analysis identified several overlapping sets of genes involved in biosynthetic processes, monocarboxylic acid metabolism, lipid metabolic processes, and the regulation of lipid metabolism (Figure 4B). The genes involved in fatty acid metabolism that were upregulated in the atria of hyperoxia-exposed mice relative to controls included peroxisome proliferator–activated receptor γ coactivator 1-α (*Ppargc1α*), a transcriptional coregulator that binds to peroxisome proliferator–activating receptor α and other transcription factors to promote mitochondrial biogenesis (35). Hyperoxia also upregulated acetyl-CoA carboxylase 2 (*Acacb*), a mitochondrial localized isoform of acetyl-CoA carboxylase that produces malonyl-CoA for mitochondrial biogenesis and regulation of β-oxidation (36), hydroxyacyl-CoA dehydrogenase trifunctional multienzyme complex subunit α (*Hadha*), part of the mitochondrial complex that catalyzes the β-oxidation of long-chain fatty acids (37), and lipoprotein lipase (*Lpl*), which cleaves lipids from dietary lipoproteins (38).

Neonatal hyperoxia reduced expression of several other genes involved in fatty acid metabolism. These included the core enzymes in the de novo fatty acid synthesis pathway, such as solute carrier family 25 member 1 (*Slc25a1*), the channel that transports citrate produced by the TCA cycle out of the mitochondria and into the cytoplasm (39, 40); ATP-citrate lyase (*Acly*), the enzyme that converts cytoplasmic citrate to acetyl-CoA (41); and acetyl-CoA carboxylase 1 (*Acaca*), the cytoplasmic isoform of acetyl-CoA carboxylase that produces malonyl-CoA for fatty acid synthesis (42). In addition, hyperoxia reduced mRNA for *fatty acid synthase* (*Fasn*),the enzyme that synthesizes the 16-carbon saturated fatty acid palmitate from acetyl-CoA and malonyl-CoA (43); *elongation of very long chain fatty acids 6* (*Elovl6*), the enzyme that extends palmitate to produce the 18 carbon saturated fatty acid stearate (44); *stearoyl-CoA desaturase 1* (*Scd1*), which converts palmitate and stearate to the mono-unsaturated fatty acids palmitoleate and oleate (45); and *thyroid hormone-inducible hepatic protein* (*Thrsp*), which binds FASN and increases its activity (46). Neonatal hyperoxia also downregulated cell death inducing *DFFA like effector c* (*Cidec*), a protein required for the storage of triglycerides as lipid droplets (47), and *adiponectin* (*Adipoq*), an adipokine that protects cardiomyocytes from oxidative stress and apoptosis following ischemic injury (48).

The reduced expression of fatty acid synthesis genes in the atria of mice that were exposed to neonatal hyperoxia was intriguing, since this pathway is required for the proliferation and survival of both cancerous and noncancerous cells (49–53). The expression of fatty acid synthesis genes was, thus, examined in an independent set of mice to confirm whether neonatal hyperoxia suppresses their expression in the atria. Quantitative PCR (qPCR) showed that *Slc25a1*, *Acly*, *Acaca*, *Fasn*, *Scd1*, and *Thrsp* were all expressed at lower levels in the atria of P4 mice exposed to neonatal hyperoxia mice compared with those of controls (Figure 4C). Mean expression of Elovl6 was also lower in hyperoxia-exposed mice relative to controls, but the *P* value for this change was above the threshold set for significance (*P* = 0.065). In contrast, none of these genes were significantly affected in the ventricles on P4 (Figure 4D). *Acacb* was examined in the hearts of hyperoxia-exposed mice, but the increased expression in our microarray was not significant in the atria or ventricles of hyperoxia-exposed mice by qPCR. Since mice exposed to hyperoxia are returned to room air on P4, we investigated when expression of these genes returned to control levels. Surprisingly, mRNA for *Slc25a1*, *Acly*, *Acaca*, *Fasn*, *Scd1*, *Thrsp*, and *Elovl6* remained suppressed on P56 (Figure 4E). These changes were again specific for the atrium, and the expression of *Slc25a1, Acly, Acaca, Acacb, Thrsp, Fasn,* and *Scd1* in the ventricle was unaffected on P56, while *Elovl6* was not detected (Figure 4F). Hyperoxia, thus, permanently suppresses fatty acid synthesis genes in the atria but not ventricles.

**Figure 5:**
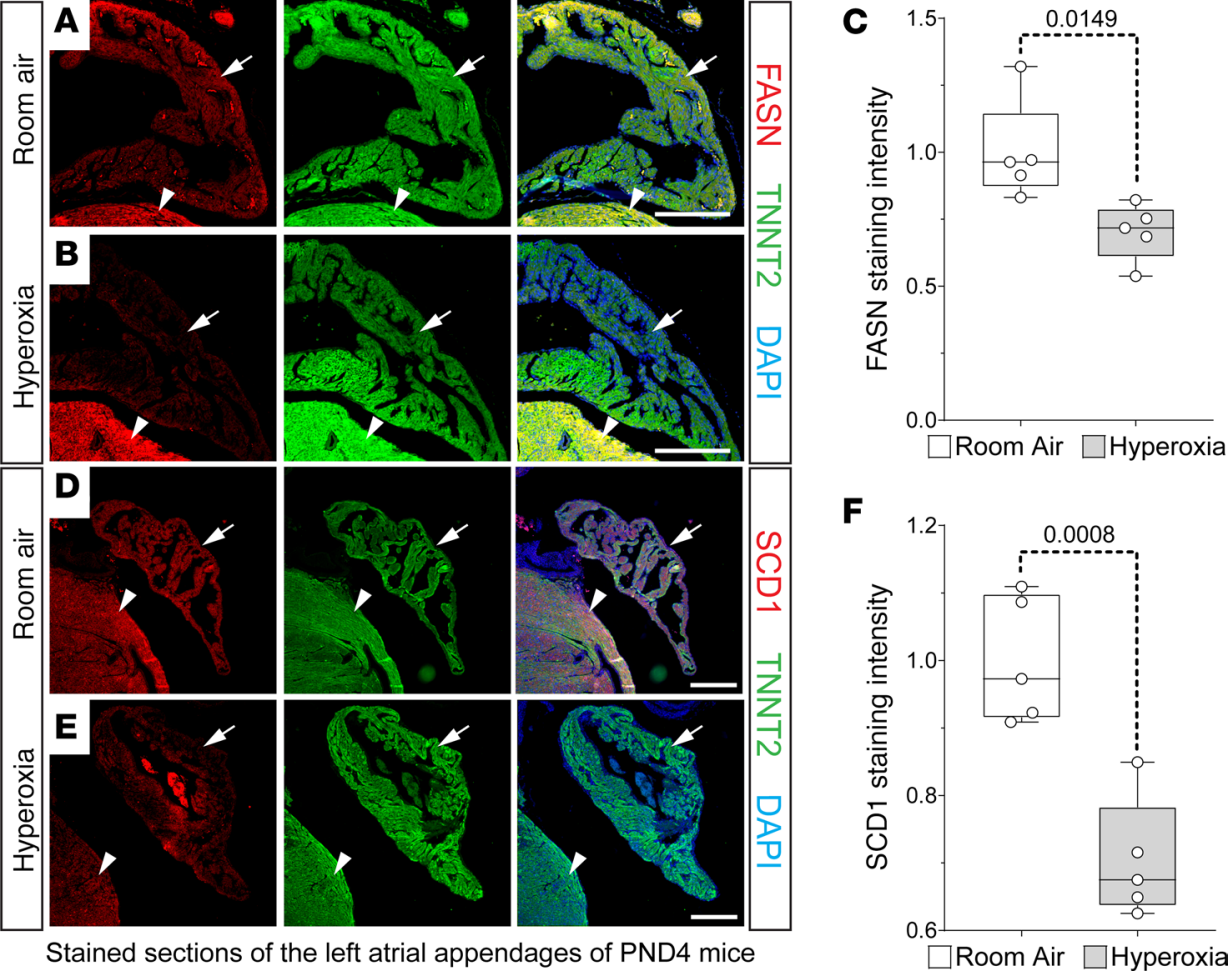
Neonatal hyperoxia represses fatty acid synthesis enzymes in murine atrial cardiomyocytes. (**A**, **B**, **D**, and **E**). Sections of left atrial appendages from P4 neonates exposed to room air (**A** and **B**) or hyperoxia (**D** and **E**) costained for FASN (red, **A** and **B**) or SCD1 (red, **D** and **E**) and TNNT2 (green, **A**, **B**, **D**, and **E**). Sections were also stained with DAPI to label nuclei (blue, **A**, **B**, **D**, and **E**). Arrows and arrowheads show TNNT2^+^ cardiomyocytes in left atrial appendage and LV, respectively. Scale bars: 200 μm. (**C** and **F**) Graphs show relative staining intensities for *Fasn* (**C**) and *Scd1* (**F**) measured using NIH ImageJ 2.0/Fiji. (**C** and **F**) Room air, *n* = 5; hyperoxia, *n* = 5. Box plots show median values and inner quartiles. Whiskers show the range of values. Circles show values for individual room air– and hyperoxia-exposed mice, respectively. *F* tests were used to determine if samples had equal or unequal variances. *P* values are the results of unpaired 2-tailed *t* tests.

IHC was used to confirm that neonatal hyperoxia inhibited FASN and SCD1 protein in left atrial cardiomyocytes. Staining for FASN (red; Figure 5, A and B) and TNNT2 (green; Figure 5, A and B) detected FASN in the atrial (arrows) and ventricular (arrowheads) cardiomyocytes of P4 mice exposed to room air. In contrast, FASN staining was reduced in the left atrial cardiomyocytes of hyperoxia-exposed mice (Figure 5C) but unaffected in ventricular cardiomyocytes. Staining for SCD1 (red; Figure 5, D and E) and TNNT2 (green; Figure 5, D and E) showed that SCD1 was similarly reduced in left atrial cardiomyocytes of P4 mice exposed to neonatal hyperoxia relative to controls (arrows; Figure 5, D–F). As observed for FASN, SCD1 levels were less affected in the ventricles of hyperoxia-exposed and room air control mice (arrowheads; Figure 5, D and E).

### Hyperoxia inhibits fatty acid synthesis genes and proliferation in the HL-1 line of immortalized murine atrial cardiomyocytes.

**Figure 6:**
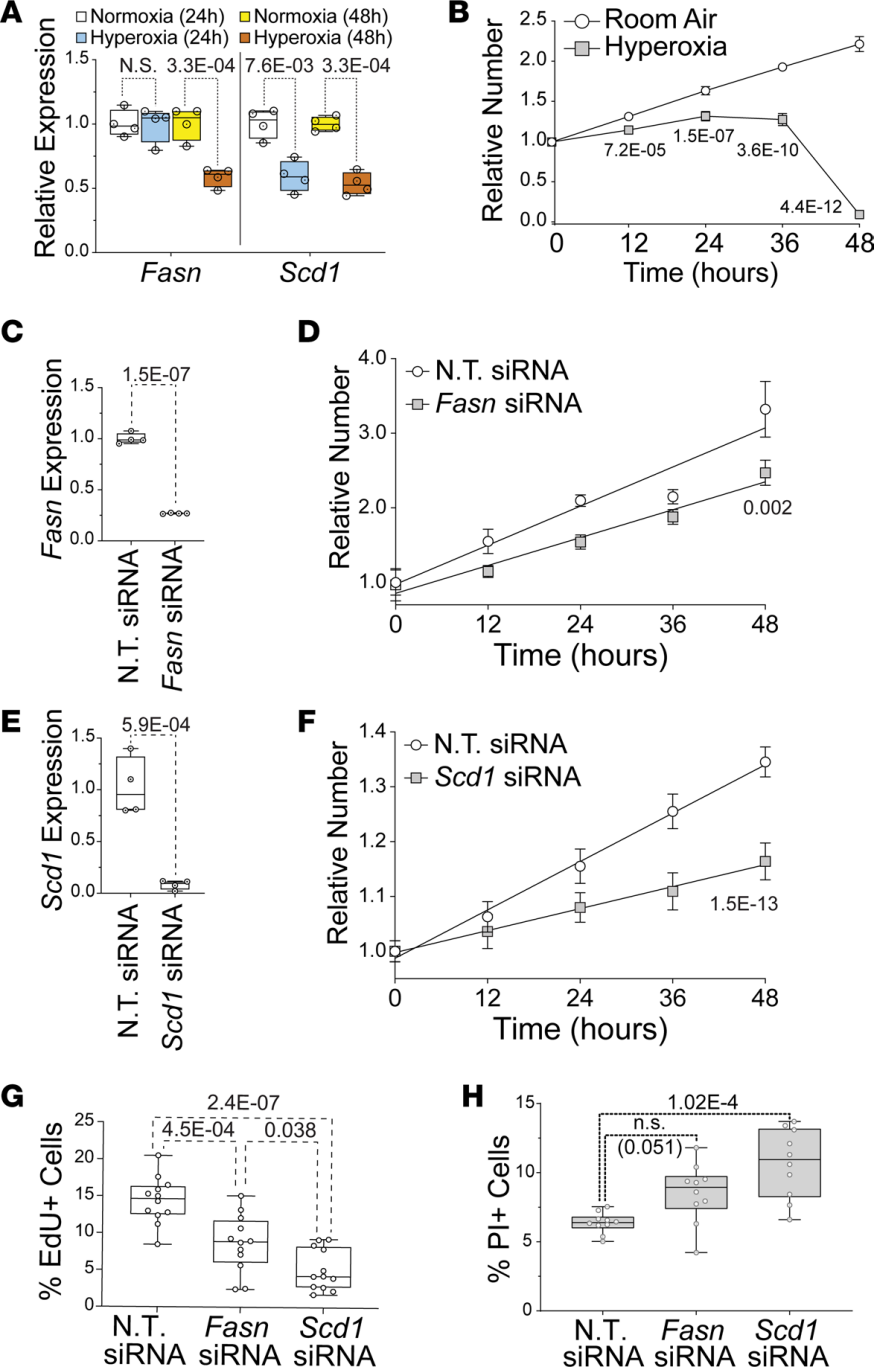
Suppression of fatty acid synthesis contributes to reduced HL-1 cell proliferation in hyperoxia. (**A**) qPCR for *Fasn* (left) and *Scd1* (right) in HL-1 cells grown in room air or hyperoxia for 24 hours (white and blue, respectively) or 48 hours (yellow and red, respectively). (**B**) Numbers of HL-1 cells grown in room air (white circles) and hyperoxia (gray squares) for 48 hours relative to their density at 0 hours. (**C** and **E**) qPCR for *Fasn* (**C**) and *Scd1* (**E**) in HL-1 cells transfected with *Fasn* and *Scd1* siRNAs. Controls were transfected with nontargeting (NT) siRNA. (**D** and **F**) Numbers of HL-1 cells transfected with control siRNA (white circles in **D** and **F**) and *Fasn* (Gray squares in **D**) or *Scd1* (Gray squares in **F**) siRNAs and grown for 48 hours in room air relative to 0 hrs. (**G**) HL-1 cells transfected with NT, *Fasn*, and *Scd1* siRNAs, grown in room air for 22 hours and treated with EdU for 2 hours before staining. Graph shows percentages of EdU^+^ cells. (**H**) HL-1 cells transfected with NT, *Fasn*, and *Scd1* siRNAs were grown in room air for 48 hours, stained with PI, and imaged. Graph shows numbers of PI^+^ NT, *Fasn,* and *Scd1* siRNA transfected cells. (**A**, **C**, and **E**) *n* = 4 transfections per condition. Each time/condition: (**G** and **H**), *n* = 12. (**B**, **D**, and **F**) Error bars show 95% CI; lines and *P* values are results of linear regressions. (**A**, **C**, **E**, **G**, and **H**) Box plots are median, second quartiles, and third quartile; whiskers indicate range, and markers show individual replicates. *P* values are from unpaired 2-tailed *t* tests (**C** and **E**) or 1-way ANOVA with Holm-Sidak corrections (**A**, **G**, and **H**).

The HL-1 line of immortalized atrial cardiomyocytes was used to examine the direct effects of hyperoxia on fatty acid synthesis genes (54). Cells were grown in room air (21% O_2_, 5% CO_2_) or hyperoxia (95% O_2_, 5% CO_2_) for 24 or 48 hours before *Fasn* and *Scd1* mRNA were quantified by qPCR (Figure 6A). Relative to cells grown in room air, *Fasn* and *Scd1* were reduced in HL-1 cells after 48 hours in hyperoxia. Interestingly, while *Scd1* was lower in HL-1 cells after 24 hours of hyperoxia, *Fasn* was unaffected. However, this difference in timing is unlikely to reflect a regulatory hierarchy between genes because *Scd1* knockdown does not affect *Fasn* expression (our unpublished observation). Hyperoxia can, thus, directly affect fatty acid synthesis genes in atrial cardiomyocytes without the need for other cell types.

To determine if the suppression of fatty synthesis genes in hyperoxia-treated HL-1 cells correlates with reduced proliferation, asynchronously dividing HL-1 cells were plated at equal density and cultured in room air or hyperoxia for 0, 12, 24, 36, and 48 hours before being stained with DAPI and counted. HL-1 cells expanded continuously for 48 hours in room air (Figure 6B). In contrast, HL-1 cells grown in hyperoxia expanded slower than controls for the first 24 hours, plateaued from 24 to 36 hours, and then declined in numbers from 36 to 48 hours. These data indicate that hyperoxia directly represses proliferation and fatty acid synthesis genes in HL-1 cells, as it does in the atria of mice.

### Fasn and Scd1 are required for the proliferation and survival of HL-1 atrial cardiomyocytes.

To determine if fatty acid synthesis is required for atrial cardiomyocyte proliferation, HL-1 cells were transfected with nontargeting (NT) siRNA and siRNA for *Fasn* or *Scd1* and grown in room air for 72 hours. qPCR revealed that *Fasn* and *Scd1* mRNA were reduced around 75% and 90% in cells transfected with *Fasn* and *Scd1* siRNA, respectively, when compared with NT siRNA transfected controls (Figure 6, C and D). Asynchronously dividing *Fasn, Scd1*, and NT siRNA–treated HL-1 cells were then plated at equal numbers and counted at 12-hour intervals (Figure 6, E and F). Cells transfected with *Fasn* and *Scd1* siRNA grew slower than NT siRNA transfected controls over 48 hours.

To confirm whether *Fasn* and *Scd1* are required for HL-1 cell proliferation, cells were treated with DMSO as a vehicle control, the FASN inhibitor G28UCM at 10 μM, or the SCD1 inhibitor A939572 at 10 nM (Tocris Biosciences) and grown in room air (Supplemental Figure 1). While cells in control media grew continuously for 72 hours, cells in A939572 plateaued after 24 hours and cells in G28UCM grew slower than controls during the first 24 hours before declining in number from 24 and 72 hours. To specifically examine the effects of *Fasn* and *Scd1* knockdown on proliferation, *Fasn, Scd1*, and NT siRNA transfected cells were grown for 23 hours, treated with EdU for 1 hour, fixed, and stained with an anti-EdU antibody and DAPI. The fraction of EdU^+^ cells was reduced in *Fasn* and *Scd1* siRNA–treated cells relative to controls (Figure 6G). Propidium iodide (PI) exclusion assays were performed to determine if *Fasn* and *Scd1* were required for HL-1 cell survival. The percentage of PI^+^ cells was significantly higher in *Scd1* siRNA transfected cells than controls. The percentage of PI^+^ cells was also higher in cells transfected with *Fasn* siRNA than controls, but the *P* value of this change was slightly below the threshold for statistical significance (*P* = 0.051; Figure 6H). Together, these data show that the *Fasn and Scd1* are required for atrial cardiomyocyte proliferation and survival.

### Fasn and Scd1 overexpression restores proliferation of HL-1 atrial cardiomyocytes in hyperoxia.

**Figure 7:**
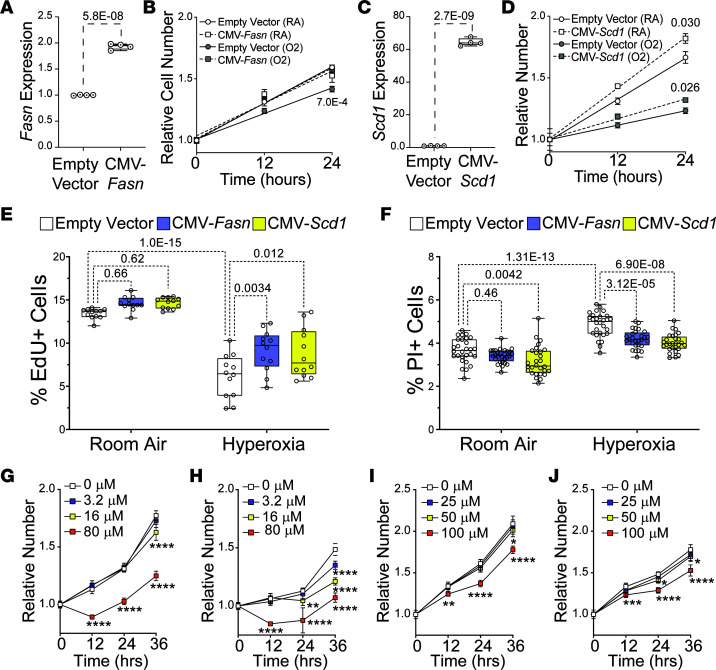
*Fasn* and *Scd1* overexpression increases HL-1 cell proliferation in hyperoxia. (**A** and **C**) *Fasn* (**A**) and *Scd1* (**C**) mRNA in HL-1 cells 48 hours after transfection with empty vector or *Fasn* (**A**) and *Scd1* (**C**) expression vectors. *n* = 4 transfections per condition. (**B** and **D**) Expansion of *Fas*n (**B**), *Scd1* (**D**), and control transfected HL-1 cells over 24 hours. Markers are mean fold change in cell number for control (circles), *Fasn* (squares with dashed line, **B**), and *Scd1* (squares with dashed line, **D**) grown in room air (white) or hyperoxia (gray). Lines and *P* values are linear regressions. *n* = 10 wells per time/condition. (**E**) Percentages of EdU^+^ control (white), *Fasn* (blue), and *Scd1* (yellow) transfected HL-1 cells after 2 hours incubation in room air (left) and hyperoxia (right). *n* = 12 wells per condition. (**F**) Percentages of control (white), *Fasn* (blue), and *Scd1* (yellow) transfected cells labeled after PI staining in room (left) and hyperoxia (right). *n* = 28 wells per condition. (**G** and **H**) HL-1 cell expansion in media with 0, 3.2, 16, and 80 μM palmitate-BSA in room air (**G**) or hyperoxia (**H**) for 36 hours. (**I** and **J**) Expansion of HL-1 cells in media containing 0, 25, 50, and 100 mM oleate-BSA and grown in room air (**I**) or hyperoxia (**J**) for 36 hours. (**A**, **C**, **E**, and **F**) Box plots show median, second quartiles, and third quartiles; markers represent individual values; whiskers show range. *P* values are results of unpaired 2-tailed *t* tests (**A** and **C**) or 1-way ANOVA with Holmes-Sidak corrections. (**B** and **D**) Trendlines and *P* values linear regressions. (**G**, **H**, **I**, and **J**) *n* = 18 wells per time/condition. *P < 0.05, **P < 0.01, ***P < 0.005, ****P < 0.001 using 2-way ANOVA with Tukey’s multiple comparison tests.

We next sought to determine how *Fasn* and *Scd1* overexpression would affect atrial cardiomyocyte proliferation in hyperoxia. Cells were transfected with *Fasn* and *Scd1* expression vectors (*CMV-Fasn* and *CMV-Scd1*, respectively), as well as empty expression vector. After 24 hours, cells were replated at equal density and grown in room air or hyperoxia for 0, 12, and 24 hours before being stained and counted. Cells transfected with *CMV-Fasn* expressed approximately 2-fold more *Fasn* than empty vector transfected cells (Figure 7A). As expected, control transfected cells grew slower in hyperoxia than in room air (Figure 7B). However, *CMV-Fasn* transfected cells grew at the same rate as control cells grew in room air, regardless of whether they were in room air or hyperoxia. Cells transfected with *CMV-Scd1* expressed approximately 60-fold more *Scd1* than controls (Figure 7C) and grew faster than control cells in room air and hyperoxia (Figure 7D). *Fasn* and *Scd1* overexpression, thus, partially restores HL-1 cell growth in hyperoxia.

To specifically examine the effects of *Fasn* and *Scd1* overexpression on proliferation, HL-1 cells were transfected with control, *Fasn*, and *Scd1* plasmids; grown in room air or hyperoxia for 23 hours; and treated with EdU to label cells in S-phase (Figure 7E). While numbers of EdU^+^ cells were unaffected by *Fasn* and *Scd1* overexpression in room air, more *Fasn*- and *Scd1*-overexpressing cells were EdU^+^ in hyperoxia than controls. Cells transfected with control, *Fasn*, and *Scd1* plasmids were also grown in room air or hyperoxia for 48 hours and subject to PI exclusion assays to determine if *Fasn* and *Scd1* promote survival in hyperoxia (Figure 7F). Consistent with the increased numbers of cl-CASP3^+^ cells in the atria of P4 hyperoxia-exposed mice, 48 hours of hyperoxia increased the percentage of control transfected cells stained by PI. While *Scd1* overexpression caused a modest reduction in the numbers of PI^+^ cells in room air, *Fasn* did not. However, *Fasn-* and *Scd1*-overexpressing cells were both less likely to be PI^+^ in hyperoxia than controls. Restoring *Fasn* and *Scd1* expression can, thus, partially restore the survival and proliferation of atrial cardiomyocytes in hyperoxia.

If *Fasn* and *Scd1* promote the growth of HL-1 cells in hyperoxia by increasing the supply of their products, adding exogenous fatty acids to the culture media may mimic their effects. Cells were, thus, plated in media containing increasing amounts of BSA-conjugated palmitate (Figure 7, G and H) or oleate (Figure 7, I and J) and grown in room air (Figure 7, G and I) or hyperoxia (Figure 7, H and J) for 36 hours. In room air, cells grew at equal rates in media containing low and medium levels of palmitate and oleate conjugated BSA as they did in media containing unconjugated BSA. In contrast, high concentrations of palmitate and oleate slowed the growth of cells in room air relative to controls. High levels of palmitate and oleate similarly slowed HL-1 cell growth in hyperoxia relative to controls. As seen in room air, low and medium concentrations of oleate did not affect HL-1 cell growth in hyperoxia. However, HL-1 cells treated with low and intermediate levels of palmitate grew slower in hyperoxia than cells in control media, suggesting that hyperoxia enhances the effects of palmitate-BSA on their expansion. Palmitate and oleate are, thus, not sufficient for the effects of *Fasn* and *Scd1* overexpression on atrial cardiomyocyte growth in hyperoxia.

### Hyperoxia does not reduce SREBF1 protein in atrial cardiomyocytes.

Genes in the de novo fatty acid synthesis pathway are regulated by sterol-response element binding factor 1 (SREBF1), a transcriptional master regulator of lipogenesis (55). HL-1 cells were, thus, transfected with plasmid that expresses human SREBF1 under the control of the *CMV* promoter (*CMV-Srebf1*) or empty vector and grown in hyperoxia or room for 36 hours. Performing qPCR with a primer pair recognizing both human and mouse *Srebf1* mRNA showed that cells transfected with *CMV-Srebf1* expressed nearly twice as much *Srebf1* compared with control cells (Supplemental Figure 2A). *CMV-Srebf1* transfected cells grew slower in room air than those transfected with empty vector (Supplemental Figure 2B). However, while control transfected cell growth arrested after 12 hours in hyperoxia, *CMV-Srebf1* transfected cells continued proliferating in hyperoxia up to 36 hours (Supplemental Figure 2C).

To determine if hyperoxia suppresses fatty acid synthesis genes by reducing SREBF1 protein, Western blotting for SREBF1 was performed on HL-1 cells grown in room air or hyperoxia for 48 hours (Supplemental Figure 2D; see supplemental material for unedited blots). Two bands of SREBF1 protein were detected in hyperoxia- and control-treated HL-1 cells, a 120 kd band representing full-length inactive SREBF1 within the golgi membrane, and a 55 kd band representing the amino-terminus of SREBF1, which localizes to the nucleus after cleavage. Despite the reduced expression of *Fasn, Scd1*, and other fatty acid genes in hyperoxia-treated cells, hyperoxia does not alter the levels of either band of SREBF1 protein (Supplemental Figure 2, F and G). Lysates of left atria from individual P4 hyperoxia-exposed and control mice were also Western blotted for SREBF1 protein (Supplemental Figure 2E; see supplemental material for unedited blots). Surprisingly, despite having lower levels of fatty acid synthesis genes than controls, the left atria of P4 hyperoxia-exposed mice had higher levels of both forms of SREBF1 than controls (Supplemental Figure 2, H and I). Therefore, hyperoxia is unlikely to reduce fatty acid synthesis in atrial cardiomyocytes by reducing SREBF1 protein in these cells.

### Hyperoxia inhibits fatty acid synthesis genes and proliferation in human left atrial tissue explanted from infant donors.

**Figure 8:**
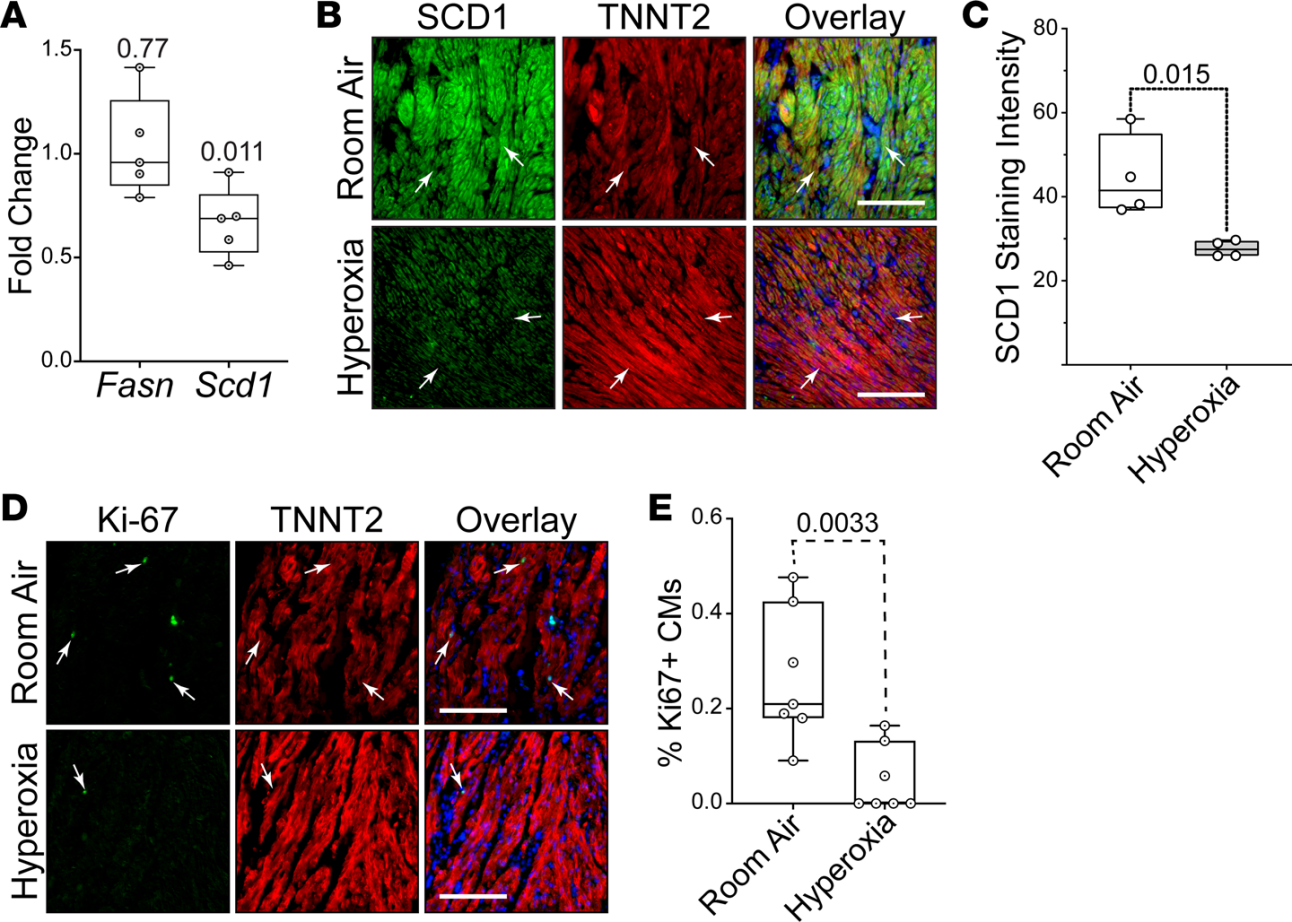
Hyperoxia suppresses fatty acid synthesis genes in left atrial tissue explanted from human infants. (**A**) Results of qPCR for *Fasn* and *Scd1* in left atrial tissue explanted from human infants who died at birth due to anencephaly and exposed to room air or hyperoxia for 24 hours. *n* = 5 donors. (**B**) Sectioned explants exposed to room air (top) or hyperoxia (bottom) were stained for SCD1 (green), TNNT2 (red), and DAPI (blue). (**C**) Graph shows staining intensities for SCD1 in sections of control and hyperoxia-exposed mice determined using NIH ImageJ 2.0/Fiji. *n* = 4 donors. (**D**) Sections of explants exposed to room air (top) and hyperoxia (bottom) stained for the proliferation marker Ki67 (green), TNNT2 (red), and DAPI (blue). (**E**) Graph shows percentages of TNNT2-expressing cells with Ki67^+^ nuclei in explants exposed to room air or hyperoxia. *n* = 7 donors. (**A**, **C**, and **E**) Circles indicate individual values for explants of each donor. Boxes show medians and inner quartiles; whiskers represent the range. *P* values are the results of either single-sample 1-tailed *t* test and Wilcox test (**A**) or unpaired 2-tailed *t* tests (**C** and **E**). (**B** and **D**) Scale bars = 100 μm.

To determine if changes observed in mice occur in humans, left atrial tissue from term infants who died shortly after birth due to anencephaly was obtained from the Biorepository for Investigation of Neonatal Diseases of the Lung (BRINDL) at the University of Rochester. The muscle was separated from surrounding tissue, cut into ~1 mm^3^ cubes, grouped, and cultured in room air or hyperoxia for 24 hours before being used for qPCR or immunostaining. While hyperoxia did not alter the levels of *Fasn* mRNA, *Scd1* expression was lower in hyperoxia-exposed explants than controls (Figure 8A). Immunological staining showed that SCD1 protein localized to TNNT2^+^ cardiomyocytes in explants exposed to room air but not in those exposed to hyperoxia (arrows; Figure 8, B and C). Sectioned explants were stained for the proliferation marker Ki-67 and TNNT2 to determine if hyperoxia reduced the proliferation of human left atrial cardiomyocytes. Ki67^+^ nuclei were more frequently observed in the TNNT2^+^ cardiomyocytes of control explants than in those exposed to neonatal hyperoxia (arrows; Figure 8, D and E). Hyperoxia, thus, suppresses fatty acid synthesis genes and proliferation in human left atrial cardiomyocytes, as it does in mice.

## Discussion

There is a significant need to understand why high oxygen exposure at birth is a risk factor for adult cardiovascular disease. We previously showed that neonatal hyperoxia causes pulmonary hypertension in adult mice and that this phenotype was preceded by a loss of the cardiomyocytes surrounding the pulmonary vein due to reduced proliferation (29). We now extend these findings by showing that hyperoxia similarly inhibits the postnatal proliferation of left atrial cardiomyocytes and causes the myocardium of this chamber to be hypoplastic. Despite having fewer cardiomyocytes per unit area, the left atria of hyperoxia-exposed mice were larger than controls, suggesting that the remaining cardiomyocytes hypertrophy to compensate for reduced numbers. Pathology was first seen on P56 when most of the hyperoxia-exposed mice examine had E/A ratios > 2. By P365, the mean E/A ratio was higher in mice exposed to neonatal hyperoxia than controls. The velocity of flow across the mitral valve during atrial contraction was also lower in hyperoxia-exposed mice than controls, suggesting that hyperoxia reduced left atrial contractility by this age. Interestingly, the 2 mice with the highest E/A ratios (>5) had left ventricles that were larger than controls during both systole and diastole. The left ventricular dilation in these mice may be adaptive since it would raise cardiac output in the presence of diastolic dysfunction, reduced fractional shortening, and lower ejection fraction. In support of this idea, excluding the 2 mice with the highest E/A ratios from our analyses, diastolic volume, stroke volume, and cardiac output were lower in the remaining hyperoxia-exposed mice than controls. Mice exposed to hyperoxia during early postnatal life, thus, develop diastolic dysfunction and heart failure as adults.

Affymetrix arrays, qPCR and IHC revealed that neonatal hyperoxia inhibited genes needed for fatty acid synthesis in the atrial but not ventricular cardiomyocytes of mice. These included *Fasn* and *Scd1*, which are elevated in highly proliferative tumors, sufficient to drive the expansion of noncancerous cells when overexpressed and required for the proliferation and survival of noncardiac cells (49, 53, 56–60). We thus explored whether the repression of *Fasn* and *Scd1* is responsible for the reduced proliferation of left atrial cardiomyocytes in mice exposed to neonatal hyperoxia. Left atrial cardiomyocytes can be isolated from neonatal mice, but the yield is low, and the resulting cells lose their proliferative capacity quickly in culture, making it difficult to examine how genetic manipulations affect their expansion. The HL-1 line of immortalized atrial cardiomyocytes was, thus, chosen to model the effects of hyperoxia on neonatal atrial cardiomyocytes, since these cells are differentiated and contractile but still metabolically immature and proliferative (54, 61, 62). Consistent with the loss of atrial CM proliferation and survival in hyperoxia-exposed mice, exposing HL-1 cells to hyperoxia for 48 hours suppressed *Fasn* and *Scd1* and reduced proliferation and survival. Inhibitors and siRNAs for *Fasn* and *Scd1* also reduced HL-1 cell proliferation and survival, while overexpressing these genes partially restored growth in hyperoxia. The repression of *Fasn* and *Scd1* may, therefore, mediate the inhibitory effects of neonatal hyperoxia on the postnatal proliferation and survival of left atrial cardiomyocyte.

Surprisingly, adding palmitate and oleate to the culture media was not sufficient to reproduce the effects of *Fasn* and *Scd1* overexpression on the expansion of HL-1 cells in hyperoxia. It is possible that fatty acids produced de novo are preferred over exogenous fatty acids for synthesizing phospholipids and other components of new membranes for dividing cells. Alternatively, fatty acid synthesis may prevent potentially toxic metabolic precursors from accumulating. Chemical inhibition and siRNA-mediated knockdown of FASN inhibits oxidative phosphorylation in immortalized melanocytes (49). This loss of mitochondrial function was partially due to the accumulation of malonyl-CoA, which inhibits the transport of fatty acids into mitochondria at high concentrations. FASN inhibition also reduces mitochondrial membrane potential (ΔΨm) and increases production of ROS (63, 64). SCD1 inhibition similarly disrupts mitochondrial function, increases ROS, and induces apoptosis in many cells, including neonatal ventricular cardiomyocytes (65, 66). Reduced SCD1 function also activates AMP-dependent protein kinase (AMPK) in many contexts, including the hearts of *Scd1*-KO mice (45, 67, 68). AMPK phosphorylates and inhibits the pro-proliferative kinase mTOR and, thus, inhibits tumor cell proliferation and survival (69). AMPK also reduces fatty acid synthesis through inhibitory phosphorylation of ACACA, as well as SREBF1 (70), and may play a role in the hyperoxia-induced suppression of fatty acid synthesis in atrial cardiomyocytes.

Recent echocardiographic imaging, MRI, and CT imaging studies indicate that preterm infants have larger left atria and lower diastolic function than term infants (14–17), suggesting that the heart failure among young adults born preterm has developmental origins. Serum from preterm infants was shown to have higher malonyl-carnitine and lower palmitoyl-carnitine levels than serum from term infants (71), suggesting that preterm birth and reduced FASN activity may be linked. Preterm infants often require mechanical ventilation, steroids, and other interventions that make it difficult to identify direct effects of supplemental oxygen on cardiovascular health. We thus sought to determine if hyperoxia suppresses fatty acid synthesis genes and proliferation in explanted human left atrial tissue. Hyperoxia reduced *Scd1* expression and the numbers of Ki67^+^ cardiomyocytes in human atrial explants, confirming that hyperoxia represses at least 1 major component of the fatty acid synthesis pathway, as well as proliferation in human left atrial cardiomyocytes. These data suggest that the response of left atrial tissue from newborn humans to hyperoxia parallels that of mice and HL-1 cells and that reduced fatty acid synthesis underlies the cardiovascular disease in preterm infants.

Exposure to neonatal hyperoxia did not suppress fatty acid synthesis genes in the ventricles of mice as it did in the atria. Since the left atrium is the first chamber to encounter oxygen-rich blood from the lungs, left atrial cardiomyocytes may have evolved to have a different response to hyperoxia than ventricular cardiomyocytes. The levels *Fasn*, *Scd1*, and other fatty acid synthesis genes were also lower in the ventricles of both hyperoxia-exposed and room air control mice at all times examined, suggesting that fatty acid synthesis may be less important for proliferation and survival of ventricular than atrial cardiomyocytes. The suppression of ventricular cardiomyocyte proliferation was hypothesized to reflect a role for oxygen in promoting their terminal differentiation and maturation (31). However, in our model, *Fasn*, *Scd1*, and other fatty acid synthesis genes continue to be suppressed in the atria of mice exposed to hyperoxia even after they are returned to room air. If hyperoxia accelerated the normal differentiation of atrial cardiomyocytes as proposed, fatty acid synthesis genes should return to similar levels in the atria of hyperoxia-exposed and control mice as the atrial cardiomyocytes of control mice matured. Exposure to hyperoxia in early neonatal life, thus, permanently reprograms atrial cardiomyocytes metabolism in a maladaptive fashion that may contribute to the long-term effects of neonatal hyperoxia on cardiovascular health. It is unclear if similar persistent molecular changes take place in ventricular cardiomyocytes.

Despite the strength of the data presented, it is important to acknowledge the limitations of our studies. HL-1 cells are a pure population of proliferative atrial cardiomyocytes that can be used to test the functional relationship between hyperoxia and fatty acid synthesis genes. However, these cells have been immortalized with the SV40 T antigen and may not faithfully recapitulate the response of primary atrial cardiomyocytes to hyperoxia or the complex interactions between cells within the left atrium. The left atrial tissue used to demonstrate that hyperoxia affects fatty acid synthesis and cardiomyocyte proliferation in humans was from anencephalic infants that were born at term and died shortly thereafter instead of preterm infants. Since oxygen sensitivity is likely to change over the course of gestation, the response of human left atrial explants may not accurately reflect how left atrial cardiomyocytes respond to supplemental oxygen in preterm infants. Moreover, hyperoxia did not suppress *Fasn* in explanted human atrial tissue as it did in the atria of mice. Since 1 day of hyperoxia was insufficient to repress *Fasn* in HL-1 cells, prolonged exposure to hyperoxia may be needed suppress *Fasn* in atrial explants. Alternatively, ex vivo studies may not recapitulate the effects of hyperoxia seen in vivo. Future studies will be required to determine if restoring fatty acid synthesis will alleviate the effects of neonatal hyperoxia on left atrial cardiomyocytes in the hearts of mice or if fatty acid synthesis genes are reduced in banked heart tissue from preterm infants.

In conclusion, the data herein suggest that exposure to hyperoxia in early postnatal life initiates the development of adult diastolic dysfunction by permanently suppressing *Fasn*, *Scd1*, and other genes needed for fatty acid synthesis, as well as cardiomyocyte proliferation and survival within the myocardia of the left atrium. Although the remaining cardiomyocytes undergo hypertrophy to compensate for the reduced numbers of cells, the reduced A-peak velocities in aged mice exposed to neonatal hyperoxia suggest that these cells lose contractility over time. Moreover, since adult cardiomyocytes use fatty acids for 70% of the ATP used for contractility (72), the long-term suppression of *Fasn, Scd1*, and other fatty acid synthesis genes may affect the functionality of the remaining cardiomyocytes after they have stopped proliferating and further contribute to the diastolic heart failure. Agonists for nuclear hormone receptors that work with SREBF1 to induce lipogenesis, such as liver X receptor (LXR) and PPARG, are currently available (73–77). Future studies will be needed to determine if these compounds can be used to protect or restore the postnatal proliferation and survival of atrial cardiomyocytes in mice exposed to hyperoxia and potentially lead to novel therapies to prevent diastolic heart failure in individuals who were born preterm.

## Methods

### Mice and hyperoxia.

C57BL/6J mice were purchased from The Jackson Laboratory and maintained as an inbred colony. Mice were exposed to humidified room air (21% O_2_) or hyperoxia (100% O_2_) between P0 and P4 (22). Some mice exposed to hyperoxia were returned to room air. Dams were cycled between room air and hyperoxia every 24 hours to reduce oxidant injury. The mice were provided food and water ad libitum and housed in microisolator cages in a pathogen-free environment. Since we have not observed differences in the response of male and female mice to hyperoxia in prior studies, male and female mice were both used in experiments performed on P4. However, all male mice were used to examine the effects of neonatal hyperoxia on cardiac function on P56 and P365 to prevent sex-specific differences in ventricular measurements from masking potential effects of hyperoxia.

### Echocardiography.

To assess cardiac function, mice were anesthetized with isoflurane and subject to transthoracic echocardiography using a VisualSonics Vevo 3100 with a 40 MHz transducer. M-mode scans along the short axis of the left ventricle were acquired at the level of the papillary muscle to determine the internal diameters and volumes of the left ventricle in systole and diastole, widths of the anterior and posterior walls of the left ventricle, mass of the free wall of the left ventricle, heart rate, fractional shortening, ejection fraction, stroke volume, and cardiac output. Doppler images of flow across the mitral valve were used to determine the Vmax during the E- and A-peaks as well as the E/A ratio. Tissue doppler images were used to determine e’ and the E/e’ ratios of mice examined on P365. Five neonatal hyperoxia-exposed and 5 control mice were examined on P56. Ten hyperoxia-exposed and 8 control mice were examined on P365. *F* tests were used to determine if the variance between values for hyperoxia-exposed and control mice had equal or unequal variance and the appropriate unpaired 2-tailed *t* test with *P* < 0.5 used to identify statistically significant differences in mean values for hyperoxia-exposed and control mice. Measurements were made by the staff of the Microsurgery and Echocardiography Core at the Aab Cardiovascular Research Institute at the University of Rochester, who were blinded to the treatment of the animals.

### Affymetrix arrays and analysis.

Total RNA was isolated from the atria of P4 mice exposed to room air or hyperoxia using Trizol (Thermo Fisher Scientific), and its integrity was validated using an Agilent Bioanalyzer (Agilent Technologies). The RNA was converted to cDNA, biotinylated with Ovation kits from NuGEN, and hybridized to the Affymetrix mouse genome 430 2.0 array. Each array was probed with RNA from an individual mouse. Arrays were stained with streptavidin-phycoerythrin as recommended. Arrays were scanned for phycoerythrin fluorescence, and spot intensities were normalized across arrays with the RMA method in the “oligo” package (78). Affymetrix cell file were loaded in R 3.4.3/Bioconductor 3.5 using the oligo package and RMA normalized. A total of 29290 features that passed filters on mean expression > 3 and total variance > 0.0025 was tested for differential expression with limma (79). A total of 157 genes with FDR-adjusted *q* < 0.30 was tested for enrichment in GO using ClusterProfiler. Complete array data sets were deposited in ArrayExpress under accession no. E-MTAB-9008 (https://wwwdev.ebi.ac.uk/gxa/experiments/E-MTAB-9008/Supplementary%20Information?accessKey=06fa8fc5-a60e-485b-a69c-3ede2c2c457d).

### qPCR.

Total RNA was isolated using Trizol, treated with DNase to remove genomic DNA, and reverse transcribed using the Maxima First Strand cDNA synthesis kit (Thermo Fisher Scientific). qPCR was performed using the primer pairs listed in Supplemental Table 5 and iTaq SYBR Green Master Mix on a CFX384 Real-Time PCR detection system (Bio-Rad). Fold changes in gene expression were calculated by the ΔΔCt method using average Ct value for housekeeping genes *Pol2ra*, and *Gapdh* to control for loading. Since data were compiled from multiple runs, sample numbers for each gene depended on availability. P4 atria and ventricle: *n* = 5 control; *n* = 6 hyperoxia-exposed except *Thrsp* and *Elovl6* in the atria, for which *n* = 7. P56 atria: *n* = 4 controls and 4 hyperoxia-exposed for *Scd1*, *Thrsp*, and *Elovl6*, while *n* = 5 hyperoxia-exposed mice for *Slc25a1*, *Acly*, *Acaca*, and *Acacb*. P56 ventricles: *n* = 5 control and 5 experimental for *Slc25a1*, *Acly*, *Acaca*, and *Acacb*, while *Fasn*, *Scd1*, *Thrsp*, and *Elovl6* were examined in *n* = 4 control and 4 hyperoxia-exposed mice. *F* tests were used to determine if the values for control and hyperoxia-exposed mice had equal or unequal variance. Unpaired 2-tailed *t* tests with *P* < 0.05 indicated significance.

### Tissue processing, histological sectioning, and immunostaining.

I.p. injections of Avertin and heparin were used to euthanize mice and prevent clotting, respectively. Hearts were perfused with PBS and 10% neutral buffered formalin (NBF) to remove blood and fix the tissue, respectively, and they were fixed in 4% PFA overnight before being embedded in paraffin, sectioned, and stained as described (20, 80). Sections were stained with H&E and Masson’s trichrome to assess morphology. The area of the left atrium was determined for 3 sections of each heart located near the aortic and mitral valves with ImageJ 2.0 (NIH)/Fiji, averaged for each individual mouse, and used as 1 biological replicate. For P4 and P56 mice, *n* = 4 controls and 4 hyperoxia exposed mice. For P365 mice, *n* = 3 controls and 4 hyperoxia exposed mice. To determine the density of cardiomyocytes within the left atrial myocardium, sections were stained for TNNT2 (Thermo Fisher Scientific, MA5-12960) and DAPI. The TNNT2^+^ areas of each atria were imaged using a Nikon E800 Fluorescence microscope (Microvideo Instruments) and a SPOT-RT digital camera (Diagnostic Instruments) so numbers of DAPI^+^ nuclei within the TNNT2^+^ areas in each image could be counted using the find maxima function of ImageJ 2.0/Fiji. For P4 mice, *n* = 4 controls and 5 hyperoxia-exposed mice were used. For P56 mice, *n* = 3 control and 3 hyperoxia-exposed mice were used. For P365 mice, *n* = 4 control and 4 control mice were used. To determine numbers of pHH3^+^ and cl-Casp3^+^ cardiomyocytes, sections were stained with for pHH3 (Cell Signaling Technology, 9701) or cl-Casp3 (Cell Signaling Technology, 9661), as well as TNNT2; imaged; and counted using ImageJ 2.0/Fiji. For pHH3 staining on P4, *n* = 4 control and 4 hyperoxia exposed mice were used. For cl-Casp3 staining on P4, *n* = 4 control and 5 hyperoxia-exposed mice were used. Antibodies for FASN and SCD1 (Thermo Fisher Scientific, PA5-19509 and PA5-17409, respectively) were used to examine protein levels and localizations in P4 mice. The intensities of FASN and SCD1 staining were determined using the measure tool of ImageJ 2.0/Fiji. *n* = 5 control and 5 hyperoxia-exposed mice for both FASN and SCD1 were used.

### Culture of immortalized murine atrial cardiomyocytes.

HL-1 cells were cultured in Claycomb media with 100 μM norepinephrine, 300 μM ascorbic acid, 10% FBS, 4 mM glutamine, and 5 units/mL penicillin and 5 µg/mL streptomycin (cells and media obtained from Sigma-Aldrich). When indicated, cells were transfected with 30 pMol of pooled siRNA against *Fasn* and *Scd1* (Horizon, L-040091-00 and M-040675-01) per well of a 6-well plate or 3.5 μg of *Fasn* and *Scd1* cDNAs (Horizon; MMM1013-202765185 and MMM1013-202762947, respectively) using Lipofectamine RNAiMax or 2000 with a 3:1 ratio of transfection reagent to nucleic acid (Thermo Fisher Scientific). Cells transfected with 30 pMol nontargeting siRNA, and 3.5 μg of empty vector served as controls. The levels of siRNA, DNA, and transfection reagents used to transfect cells in other sized cluster plates were scaled according to surface area. For growth assays, cells were plated in 96-well dishes at a density of 5000 cells/well and allowed to attach overnight. Cells were exposed to room air and hyperoxia for 48 hours, with plates being fixed at 0, 12, 24, and 48 hours. Fixed cells were stained with DAPI and scanned on a Celigo S Image Cytometer (Nexcelom Bioscience) to count nuclei/well with the associated software. When indicated, 10 mM G28UCM and 10 nM A939657 (Tocris Bioscience) were added before exposure to inhibit FASN and SCD1, respectively. Cells treated with DMSO were used as vehicle controls. For EdU incorporation, cells were exposed to room air or hyperoxia for 23 hours before 10 μM EdU was added to the media. Cells were returned to room air or hyperoxia for 1 hour and fixed before EdU^+^ cells were detected with the Click-IT EdU Cell Proliferation Kit (Thermo Fisher Scientific). Cells were costained with Hoechst and imaged on an Celigo Image Cytometer to determine percentages of EdU^+^ cells.

### Western blotting.

HL-1 cells were plated at equal density and cultured in room air or hyperoxia for 48 hours and then lysed in 2× Laemmli buffer (Bio-Rad, 161-0737) containing protease and phosphatase inhibitor cocktail (Thermo Fisher Scientific, 78442). The left atria of hyperoxia-exposed and room air control mice were dissected away from the heart using forceps and lysed in 2× Laemmli buffer with protease/phosphatase inhibitors as described. Samples were heated at 95°C for 5 minutes, separated by SDS-PAGE, and transferred onto PVDF. Membranes were stained with antibody for SREBF1 (Sigma-Aldrich, SAB2102992), HRP-conjugated goat anti-rabbit antibody (Jackson ImmunoResearch Labs, 111035045), and SuperSignal West Pico substrate (Thermo Fisher Scientific, 34080). Blots were scanned on a Bio-Rad ChemiDoc System, and densitometry was done with associated software (*n* = 3 independent wells each for room air– and hyperoxia-exposed cells; *n* = 4 left atria from control mice and 5 left atria from hyperoxia-exposed mice).

### Ex vivo culture of human left atrial tissue.

Tissue from 4 donors with anencephaly who died within 24 hours of birth at 37 weeks and 1 donor with Hirschsprung’s and demyelinating disease that died at 3 months of age was used to examine the effects of hyperoxia on *Fasn*, *Scd1*, and *Ki67* expression. Explants from these donors and another 2 donors that died after 36 weeks gestation due to anencephaly for which RNA was unavailable were sectioned and used to examine how hyperoxia affects SCD1 and Ki67 staining. In all cases, myocardium was cleaned of surrounding tissue, cut into approximately 1 mm^3^ cubes, and cultured in EMEM with Insulin-Transferrin-Selenium (ITS; Thermo Fisher Scientific). Explants were examined on an inverted microscope with heated stage to confirm they were beating and viable before and after being divided into groups of 10–15 explants and cultured in room air or hyperoxia for 24 hours. After exposure, explants were lysed for RNA extraction and qPCR or fixed for sectioning and immunologic staining. For qPCR studies, fold changes in expression between explants cultured in room air and hyperoxia were calculated for 5 donors and averaged to report mean fold changes in mRNA levels. One-sample *t* tests were used to determine if mean fold changes relative to room air exposed explants in *Fasn* and *Scd1* deviated significantly from 1.

### Statistics.

Data was analyzed with JMP12 (SAS Institute) and graphed with Prism 8 (GraphPad Software). Single variant studies were judged using unpaired 2-tailed *t* tests. *F* tests were used to determine if samples had equal or unequal variance. Simultaneously measured parameters were judged with unpaired 2-tailed *t* tests and Holm-Sidak corrections. Studies with more than 2 experimental conditions were judged using 1-way ANOVA with Tukey’s multiple comparisons tests. Growth curve and other multivariant data were judged using 2-way ANOVA with Sidak multiple comparisons tests or linear regression. In all cases, *P* < 0.05 was considered significant. The statistical analysis of Affymetrix array data and gene expression in human atrial tissue was performed as described in their subsections of the Methods section.

### Study approval.

Mouse studies were approved by the University Committee on Animal Resources at the University of Rochester (protocol no. 20070-121R). Human tissue was provided through the federal United Network of Organ Sharing via National Disease Research Interchange (NDRI) and the International Institute for Advancement of Medicine (IIAM) and was entered into the NHLBI LungMAP Biorepository for INvestigations of Diseases of the Lung (BRINDL) at the University of Rochester Medical Center, overseen by the IRB as RSRB00047606, as described (81, 82).

## Author contributions

EDC and MAO designed and supervised experiments and wrote the manuscript. GAP, GSP, PSB, and ANM helped with experimental design, data analysis, and editing. MY and EDC handled mice and collected tissues. GSP collected human samples. ANM analyzed microarray data sets. EDC, MY, and ER performed all experiments. EDC and GAP analyzed echocardiography data.

## Supplementary Material

Supplemental data

## Version 1 01/28/2021

In-Press Preview

## Version 2 03/08/2021

Electronic publication
