# Supplementary material for: Neonatal hyperoxia inhibits proliferation and survival of atrial cardiomyocytes by suppressing fatty acid synthesis
Source: JCI Insight. 2021 Mar 8;6(5):e140785. doi: 10.1172/jci.insight.140785 (PMC8021108; doi:10.1172/jci.insight.140785)
Supplement: Supplemental data [file jciinsight-6-140785-s075.pdf]

SUPPLEMENTAL DATA SECTION

This supplemental data section contains 2 figures and 4 tables for the following paper

Neonatal hyperoxia inhibits proliferation and survival of atrial cardiomyocytes by suppressing fatty acid synthesis

Ethan David Cohen[1,4]
Min Yee[1]
George A. Porter, Jr.[1]
Erin Ritzer[1]
Andrew N. McDavid[2]
Paul S. Brookes[3]
Michael A. O'Reilly[1,4]

The Departments of [1]Pediatrics, [2]Biostatistics & Computational Biology, and [3]Anesthesiology
School of Medicine and Dentistry, The University of Rochester, Rochester NY 14642

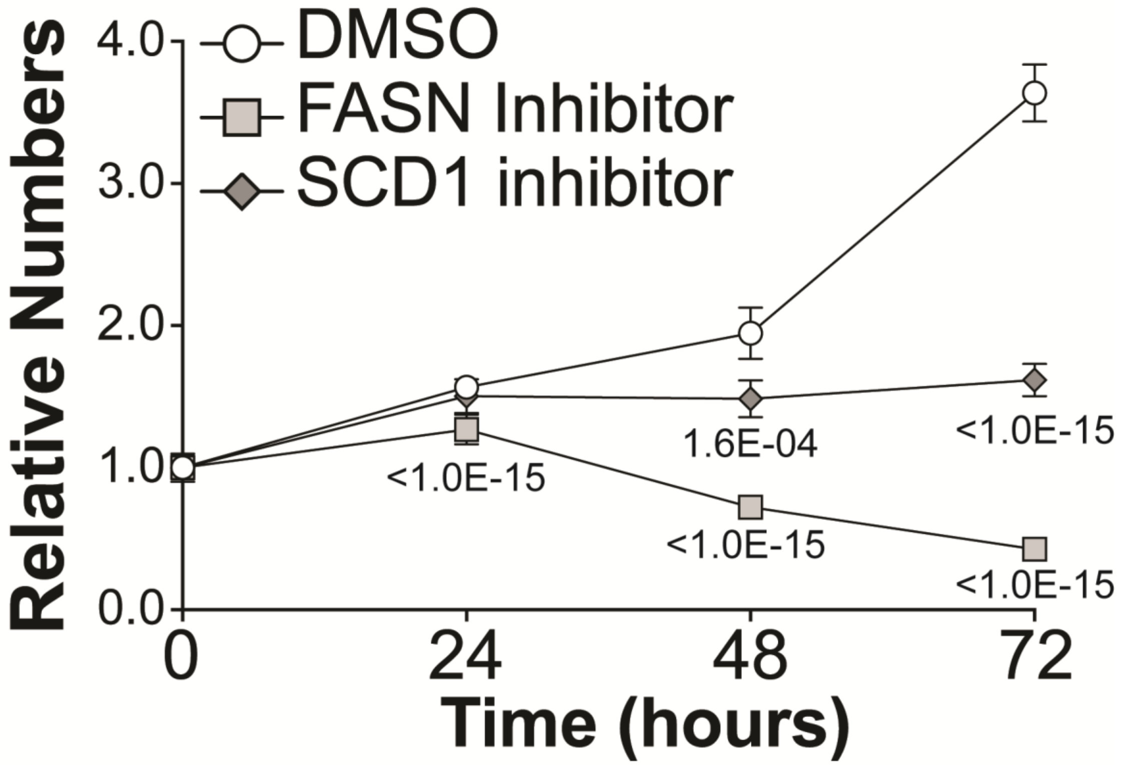

Supplemental Figure 1. Effects of FASN and SCD1 inhibitors on HL-1 cell growth. Graph shows the increase in the relative numbers of HL-1 cells grown for 72 hours in media containing DMSO (white circles), the FASN inhibitor G28UCM at 10 μM (gray squares), or the SCD1 inhibitor A939572 at 10 nM (gray diamonds). Values are normalized to number of cells in each well just prior to adding inhibitor (0hrs). The p-values shown are the results of two-way ANOVA with Dunnett’s multiple comparison tests.

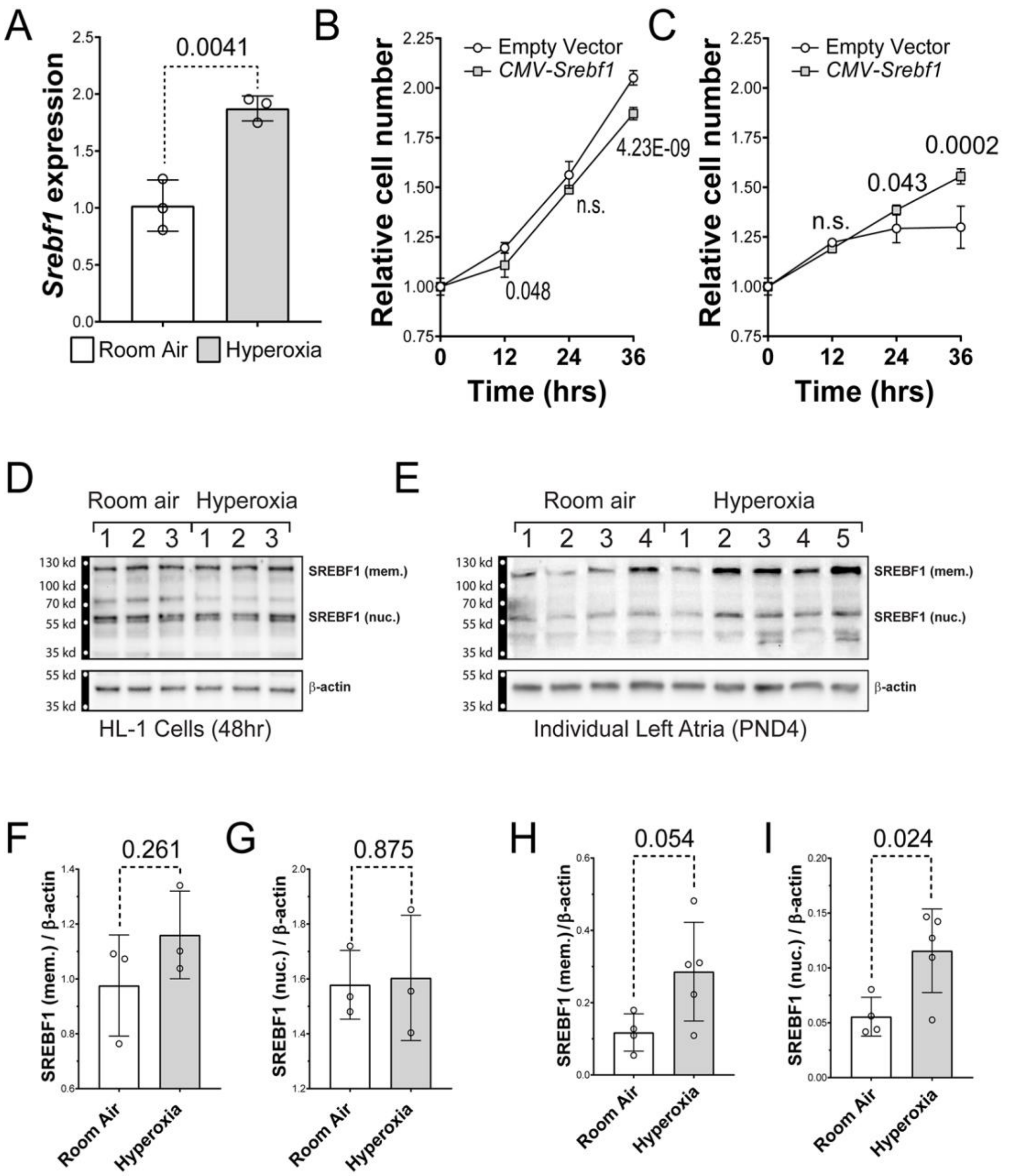

Supplemental Figure 2. SREBF1 overexpression promotes HL-1 cell expansion in hyperoxia but is not reduced in hyperoxia-exposed atria versus controls. (A) qRT-PCR for *Srebf1* in HL-1 cells transfected with empty vector or plasmid expressing human *Srebf1* using the *CMV*-promoter. Primers recognize human and mouse *Srebf1* mRNA. N=3 independent transfections per condition. (B and C) Expansion of HL-1 cells transfected with empty vector or *CMV-Srebf1* and grown in room air (B) or hyperoxia (C) for 36 hours. N=12 wells per time/condition. (D) Protein from HL-1 cells transfected with empty vector or *CMV-Srebf1* were subjected to western blotting for anti-SREBF1. The higher molecular weight band (~120kD) is membrane bound SREBF1 within the golgi while the lower molecular weight band (~55kD) shows the activated, cleaved form of SREBF1 within the cytoplasm and nucleus. β-actin antibody was used to control for loading. (E) Left atria of 4 control and 5 hyperoxia-exposed mice were lysed on PND4 and subject to western blotting for SREBF1 and β-actin. (F and G) Graphs show densitometry for membrane bound (F) and nuclear (G) SREBF1 in hyperoxia-exposed and control cells normalized to β-actin. (H and I) Graphs show the results of densitometry for the membrane bound (H) and nuclear (I) forms of SREBF1 in the individual atria of 4 control and 5 hyperoxia-exposed mice normalized to the levels of β-actin. (H, I) Room air n=4; hyperoxia n=5. (A, F, G, H, and I) Graphs show means, error bars indicate standard deviations, and circles represent individual values. p-values are results of unpaired t-tests. (B, C) White circles and shaded squares show the numbers of empty vector and *CMV-Srebf1* treated HL-1 cells relative to the numbers of cells present at time 0 for each condition. Error bars represent 95% CI, p-values shown are the results of two-way ANOVA with Tukey's multiple comparison tests.

TABLE 1. Echocardiography values of two-months old mice exposed to room air or hyperoxia as neonates

	Treatment	M1	M2	M3	M4	M5
Heart Rate In BPM	RA	419.72	518.40	389.63	523.43	424.12
	O2	576.21	562.75	568.62	561.51	576.21
Internal Diameter, Systole in mm	RA	2.77	2.38	3.39	2.37	2.90
	O2	3.02	2.20	2.47	2.43	2.41
Internal Diameter, Diastole in mm	RA	4.05	3.68	4.56	3.62	4.08
	O2	3.99	3.459	3.719	3.67	3.74
Volume; Systole in μL	RA	28.85	19.83	47.15	19.58	32.06
	O2	35.61	16.23	21.65	20.77	20.44
Volume; diastole in μL	RA	72.23	57.38	95.31	55.12	73.16
	O2	69.46	49.19	58.64	56.87	59.66
Stroke Volume in μL	RA	43.38	37.56	48.16	35.54	41.10
	O2	33.85	32.96	36.99	36.10	39.22
Ejection Fraction %	RA	60.06	65.46	50.53	64.48	56.17
	O2	48.73	67.01	63.08	63.47	65.74
Fractional Shortening %	RA	31.64	35.25	25.61	34.46	28.97
	O2	24.23	36.30	33.50	33.76	35.49
Cardiac Output in mL/min	RA	18.21	19.46	18.76	18.60	17.43
	O2	14.49	18.99	20.82	20.53	22.02
LV Mass in mg	RA	85.30	105.99	123.38	103.92	116.23
	O2	96.63	96.49	85.48	84.47	73.56
LV Mass Cor in mg	RA	68.24	84.79	98.71	83.14	92.99
	O2	77.30	77.19	68.39	67.57	58.85
LV Anterior Wall Diameter, Systole in mm	RA	1.21	1.30	0.97	1.20	1.43
	O2	1.10	1.29	1.12	1.09	1.17
LV Anterior Wall Diameter, Diastole in mm	RA	0.79	0.89	0.70	0.75	0.89
	O2	0.81	0.82	0.64	0.67	0.74

LV Posterior Wall Diameter, Systole in mm	RA	0.78	1.30	1.04	1.36	0.84
	O2	0.78	1.27	1.06	1.08	0.93
LV Posterior Wall Diameter, Diastole in mm	RA	0.45	0.76	0.71	0.90	0.66
	O2	0.58	0.83	0.74	0.72	0.48
MV A Peak Velocity in mm/s	RA	370.77	415.37	358.23	455.40	523.26
	O2	423.50	313.97	444.72	394.79	414.03
MV E Peak Velocity in mm/s	RA	713.43	772.94	642.68	720.79	806.01
	O2	729.88	506.88	1036.24	1055.91	999.03
E/A ratio	RA	1.92	1.86	1.79	1.57	1.54
	O2	1.72	1.61	2.33	2.68	2.41

Echocardiography was performed on 2-months-old mice who were exposed to room air (RA) or hyperoxia (O2) between postnatal days 0-4. The values for various cardiac measurements of 5 mice exposed to room air or hyperoxia (M1-M5) are shown.

TABLE 2. Echocardiography values of one-year old mice exposed to room air or hyperoxia as neonates

	Treatment	M1	M2	M3	M4	M5	M6	M7	M8	M9	M10
Heart Rate In BPM	RA	538.87	612.89	587.83	578.39	579.85	555.27	561.67	526.06	X	X
	O2	515.20	573.25	553.26	596.36	597.21	551.41	556.04	539.43	564.29	514.72
Internal Diameter, Systole in mm	RA	1.98	1.75	1.83	2.08	2.13	1.91	1.67	1.91	X	X
	O2	1.85	1.93	1.99	1.77	1.98	1.84	1.57	1.87	2.82	2.40
Internal Diameter, Diastole in mm	RA	3.47	3.46	3.46	3.67	3.92	3.84	3.26	3.47	X	X
	O2	3.33	3.01	3.53	3.07	3.56	3.44	2.83	2.91	4.24	4.03
Volume; systole in μL	RA	12.45	9.12	10.22	14.12	14.98	11.38	7.99	11.29	X	X
	O2	10.56	11.67	12.68	9.37	12.44	10.32	6.87	10.77	30.13	20.14
Volume; diastole in μL	RA	50.00	49.71	49.47	57.04	67.01	63.48	42.98	49.69	X	X
	O2	45.39	35.34	51.81	37.22	53.03	49.08	30.37	32.54	80.30	71.58
Stroke Volume in μL	RA	37.55	40.59	39.24	42.92	52.04	52.10	34.99	38.40	X	X
	O2	34.82	23.67	39.13	27.85	40.59	38.76	23.50	21.77	50.17	51.45
Ejection Fraction %	RA	75.06	81.66	79.30	75.25	77.60	82.05	81.44	77.26	X	X
	O2	76.78	66.84	75.43	74.69	76.62	78.92	77.43	66.89	62.46	71.84
Fractional Shortening %	RA	42.88	49.42	47.03	43.26	45.71	50.25	48.95	44.96	X	X
	O2	44.42	35.77	43.39	42.29	44.42	46.51	44.47	35.65	33.46	40.57
Cardiac Output in mL/min	RA	20.18	24.87	23.06	24.82	30.15	28.95	19.66	20.20	X	X
	O2	17.94	13.59	21.64	16.62	24.18	21.33	13.07	11.74	28.30	26.26
LV Mass in mg	RA	76.91	74.89	75.30	94.50	81.92	113.72	80.67	78.92	X	X
	O2	84.51	72.92	75.87	68.79	81.65	81.74	43.92	66.54	110.59	107.95
LV Mass Cor in mg	RA	61.53	59.91	60.24	75.60	65.54	90.98	64.54	63.13	X	X
	O2	67.60	58.34	60.70	55.03	65.32	65.39	35.13	53.23	88.47	86.36
LV Anterior Wall Diameter, Systole in mm	RA	1.17	1.20	1.15	1.20	1.09	1.45	1.15	1.16	X	X
	O2	1.26	1.16	1.27	1.25	1.10	1.28	0.99	1.17	1.16	1.24
	RA	0.70	0.77	0.70	0.85	0.75	1.04	0.88	0.81	X	X

LV Anterior Wall Diameter, Diastole in mm	O2	0.76	0.78	0.78	0.84	0.79	0.84	0.69	0.71	0.82	0.85
LV Posterior Wall Diameter, Systole in mm	RA	0.99	1.10	1.06	1.06	0.86	1.07	1.05	1.06	X	X
	O2	1.12	1.05	0.93	0.96	1.04	0.96	0.90	1.03	0.89	0.94
LV Posterior Wall Diameter, Diastole in mm	RA	0.70	0.59	0.68	0.66	0.50	0.61	0.67	0.61	X	X
	O2	0.80	0.80	0.57	0.64	0.62	0.62	0.49	0.83	0.60	0.63
MV Interventricular Septum E' in mm/s	RA	-26.11	-32.19	-14.93	-22.75	-23.45	-16.81	-20.15	-17.92	X	X
	O2	-18.53	-20.86	-21.62	-24.45	-26.84	-20.51	-28.61	-11.14	-22.83	-20.67
MV A Peak Velocity in mm/s	RA	670.40	383.18	S.P.	417.42	538.40	514.67	556.44	673.66	X	X
	O2	344.80	444.22	460.12	513.43	379.02	128.11	368.49	S.P.	123.71	144.26
MV E Peak Velocity in mm/s	RA	743.83	541.86	598.93	626.14	713.85	564.80	818.23	736.51	X	X
	O2	566.13	607.27	616.79	711.29	917.50	593.21	520.57	306.85	900.40	779.24
E/A ratio	RA	1.11	1.41	S.P.	1.50	1.33	1.10	1.47	1.093292	X	X
	O2	1.64	1.37	1.34	1.39	2.42	4.63	1.41	S.P.	7.28	5.40
IVS E/E'	RA	963.69	495.06	339.20	-27.52	-30.45	-33.59	-40.60	-41.10	X	X
	O2	450.74	570.50	592.05	613.88	-34.18	-28.92	-18.20	-27.56	-39.44	-37.70

Echocardiography was performed on one-year old mice who were exposed to room air (RA) or hyperoxia (O2) between postnatal days 0-4. The values for various cardiac measurements of 8 mice exposed to room air (M1-M8) or 10 mice exposed to hyperoxia (M1-M10) are shown. The gray highlighted values are for two oxygen-exposed mice that had higher systolic values than either control mice or the other the hyperoxia-exposed mice. S.P. Doppler imaging of flow across the mitral valve showed a single peak, making it impossible to distinguish the velocities of the E and A peaks.

TABLE 3.
List of genes whose expression increases in atria of neonatal mice exposed to hyperoxia

SYMBOL	PROBEID	log2_fc	tstat	fdr	p.value (unadjusted)	log(exp) RA	log(exp) O2
Trp53inp2	17378348	0.268	4.55	0.295	2.25E-03	6.56	6.83
Sord	17375396	0.287	4.79	0.278	1.68E-03	7.72	8.01
Acacb	17440826	0.312	5.09	0.254	1.18E-03	5.90	6.21
Actn2	17290603	0.330	4.81	0.278	1.65E-03	9.76	10.09
Pygb	17377498	0.347	4.58	0.295	2.18E-03	8.21	8.55
Kcnk3	17435811	0.353	4.70	0.287	1.88E-03	6.74	7.09
Sox18	17395928	0.363	4.78	0.278	1.71E-03	5.68	6.05
Itga9	17522916	0.364	5.88	0.192	4.86E-04	6.65	7.02
Ldhb	17472517	0.380	4.52	0.299	2.34E-03	8.74	9.12
Lyrm7	17262848	0.403	4.67	0.290	1.95E-03	5.01	5.41
Mamld1	17535284	0.403	5.27	0.242	9.52E-04	6.45	6.85
Fancf	17491417	0.420	5.38	0.231	8.49E-04	4.53	4.95
Sptb	17282025	0.425	5.10	0.253	1.17E-03	5.93	6.35
Eef1a2	17395766	0.430	5.82	0.197	5.19E-04	8.77	9.20
Nceh1	17396369	0.436	4.81	0.278	1.64E-03	6.47	6.91
Sdha	17294377	0.450	4.77	0.278	1.72E-03	9.38	9.83
Nnt	17296489	0.458	4.97	0.265	1.36E-03	7.05	7.50
Htra3	17447365	0.459	5.96	0.190	4.49E-04	8.15	8.61
Mir16-2	17398266	0.468	4.58	0.295	2.18E-03	4.29	4.76
Ppif	17297750	0.468	5.34	0.234	8.80E-04	8.38	8.85
Vsig10	17441396	0.473	4.76	0.278	1.74E-03	4.28	4.75
Gm6981	17527013	0.480	5.99	0.190	4.36E-04	6.87	7.35
Fsd2	17492810	0.488	4.94	0.268	1.41E-03	7.69	8.18
1110034G24Rik	17376608	0.495	5.11	0.253	1.15E-03	5.94	6.44
**Hadha**	**17446643**	**0.501**	**5.21**	**0.250**	**1.03E-03**	**9.62**	**10.12**
Gm11815	17423205	0.504	4.56	0.295	2.24E-03	5.43	5.94
Wdr83os	17548213	0.510	5.33	0.235	8.93E-04	9.23	9.74
Tmem82	17432247	0.512	5.91	0.191	4.74E-04	4.01	4.53
Pald1	17241206	0.514	5.73	0.204	5.71E-04	5.17	5.69
Pdgfrb	17351027	0.527	4.73	0.283	1.81E-03	6.75	7.28
Grk5	17360890	0.541	5.66	0.207	6.17E-04	6.70	7.24
Sacs	17301086	0.543	4.60	0.295	2.11E-03	4.59	5.14
**Lpl**	**17501633**	**0.548**	**5.24**	**0.250**	**9.92E-04**	**9.60**	**10.15**
Olfr1333	17429285	0.556	4.70	0.287	1.87E-03	3.65	4.20
Ephx2	17307837	0.563	5.58	0.213	6.75E-04	7.74	8.30
Slc9a3r2	17341963	0.571	4.78	0.278	1.70E-03	7.10	7.67
**Ppargc1a**	**17448001**	**0.626**	**5.69**	**0.207**	**5.97E-04**	**8.08**	**8.70**
Atp1a2	17229891	0.643	4.79	0.278	1.68E-03	7.62	8.26
Myom2	17499485	0.644	6.43	0.169	2.77E-04	7.79	8.43
Fbxo40	17330264	0.665	4.58	0.295	2.18E-03	5.62	6.28
Cdh2	17352884	0.673	6.29	0.177	3.20E-04	8.27	8.94

Acss1	17392690	0.688	6.07	0.189	3.98E-04	7.35	8.04
C330006A16Rik	17382841	0.690	4.74	0.283	1.80E-03	4.20	4.89
Cacna1c	17470235	0.692	4.79	0.278	1.68E-03	6.99	7.69
Coro6	17253258	0.733	4.50	0.299	2.42E-03	7.60	8.33
Gm24630	17445236	0.798	6.06	0.189	4.03E-04	5.40	6.20
Lrrc2	17522536	0.808	4.59	0.295	2.14E-03	7.70	8.51
Abcc9	17472536	0.865	5.76	0.204	5.53E-04	7.25	8.12
Kcnip2	17365270	0.958	4.95	0.265	1.39E-03	4.37	5.33
Gm5407	17330861	0.981	7.87	0.106	7.35E-05	3.28	4.26
Gm10663	17501516	0.993	5.11	0.253	1.15E-03	4.47	5.47
Asb15	17456414	1.112	4.51	0.299	2.39E-03	5.12	6.23
Zbtb16	17526707	1.655	8.87	0.068	3.25E-05	5.91	7.56

Total RNA was isolated from atria of postnatal day 4 mice exposed to room air or hyperoxia between PND0-4. The RNA was hybridized to the mouse genome 430 2.0 array from Affymetrix. The mean average signal intensities for each probe and the relative fold change of hyperoxia to room air were determined. The table lists those genes whose expression was significantly increased by neonatal hyperoxia as defined by a p-value < 0.05 and false discovery rate < 0.30. Genes discussed or studied in the paper are highlighted in bold.

TABLE 4.
List of genes whose expression decreases in atria of neonatal mice exposed to hyperoxia

SYMBOL	PROBEID	log2_fc	tstat	fdr	p.value (unadjusted)	log(exp) RA	log(exp) O2
NA	17404191	-2.59	-12.18	0.024	3.54E-06	7.49	4.90
Car3	17396152	-2.30	-11.98	0.024	3.97E-06	11.10	8.79
Retn	17498722	-2.26	-6.48	0.164	2.63E-04	9.07	6.80
Krt6b	17321939	-2.07	-5.04	0.260	1.25E-03	5.06	2.99
Cfd	17235018	-1.98	-7.55	0.107	9.65E-05	8.12	6.14
Krt10	17269064	-1.96	-6.65	0.161	2.22E-04	7.59	5.63
Sprr2a2	17399858	-1.94	-6.81	0.161	1.91E-04	5.33	3.40
Nr4a1	17315178	-1.93	-14.87	0.012	8.44E-07	8.48	6.56
Mup10	17425990	-1.88	-7.48	0.107	1.03E-04	5.32	3.43
Snora75	17225169	-1.86	-4.98	0.265	1.34E-03	8.08	6.22
Mup13	17426000	-1.85	-7.11	0.137	1.45E-04	5.97	4.12
Hp	17512809	-1.82	-11.93	0.024	4.09E-06	6.80	4.97
Krt2	17322026	-1.80	-4.50	0.299	2.41E-03	6.32	4.52
Krt6a	17321951	-1.79	-4.90	0.273	1.48E-03	5.52	3.73
Dsg1a	17348726	-1.74	-4.71	0.285	1.85E-03	5.62	3.88
Mup8	17425915	-1.73	-6.04	0.189	4.12E-04	5.52	3.78
Mup7	17426062	-1.72	-7.35	0.117	1.16E-04	6.11	4.40
S100a8	17399823	-1.66	-4.97	0.265	1.35E-03	4.06	2.40
**Adipoq**	**17324404**	**-1.66**	**-9.28**	**0.059**	**2.39E-05**	**6.83**	**5.17**
Krtdap	17476557	-1.63	-5.18	0.250	1.06E-03	5.65	4.02
Gm22972	17512628	-1.62	-6.76	0.161	2.01E-04	6.86	5.24
Gm21320	17426043	-1.61	-5.81	0.197	5.24E-04	5.90	4.29
Lgals12	17362342	-1.58	-10.13	0.054	1.29E-05	6.09	4.50
Cdo1	17354282	-1.57	-17.92	0.006	2.18E-07	7.38	5.81
**Scd1**	**17365098**	**-1.53**	**-10.75**	**0.042**	**8.55E-06**	**10.16**	**8.63**
**Thrsp**	**17493432**	**-1.51**	**-7.21**	**0.128**	**1.31E-04**	**8.46**	**6.95**
Mup1	17425941	-1.49	-6.33	0.176	3.07E-04	5.57	4.09
**Cidec**	**17469754**	**-1.47**	**-9.33**	**0.059**	**2.30E-05**	**6.81**	**5.34**
**Fasn**	**17273348**	**-1.44**	**-5.64**	**0.208**	**6.31E-04**	**8.18**	**6.74**
Mup2	17425900	-1.41	-6.69	0.161	2.14E-04	4.73	3.32
Tmem45b	17525288	-1.38	-7.63	0.106	9.02E-05	5.74	4.36
S100a9	17407363	-1.37	-5.08	0.254	1.20E-03	5.48	4.12
Snora74a	17349552	-1.31	-4.89	0.274	1.50E-03	7.96	6.66
Retnla	17326069	-1.23	-6.14	0.189	3.73E-04	8.13	6.90
Mup16	17426032	-1.19	-5.74	0.204	5.68E-04	4.72	3.53
Xist	17550478	-1.18	-5.10	0.253	1.17E-03	4.86	3.68
Prkar2b	17280590	-1.17	-7.80	0.106	7.81E-05	6.46	5.29
Nr4a3	17413945	-1.13	-6.59	0.161	2.37E-04	6.53	5.40
Plin1	17492406	-1.11	-7.98	0.106	6.66E-05	5.81	4.70
Slc25a1	17328829	-1.11	-6.24	0.183	3.37E-04	7.86	6.76
Inmt	17467062	-1.10	-4.73	0.283	1.81E-03	7.00	5.90

Snora31	17302054	-1.09	-5.40	0.231	8.29E-04	7.98	6.89
Snord93	17435249	-1.08	-6.54	0.162	2.49E-04	5.54	4.46
Rnu3a	17232731	-1.06	-5.36	0.231	8.59E-04	8.45	7.39
Egr1	17349514	-1.05	-4.80	0.278	1.67E-03	6.75	5.71
Serpina3c	17283675	-1.04	-9.14	0.060	2.64E-05	6.11	5.07
Car5b	17545936	-1.00	-4.61	0.295	2.10E-03	6.68	5.68
**Elovl6**	**17402558**	**-0.98**	**-6.05**	**0.189**	**4.09E-04**	**6.71**	**5.74**
Atf3	17231033	-0.97	-5.33	0.235	8.98E-04	7.15	6.18
Gm26205	17441808	-0.96	-6.35	0.176	3.01E-04	8.79	7.83
Pcx	17356216	-0.93	-4.84	0.278	1.58E-03	5.77	4.84
Klk1	17477391	-0.92	-4.62	0.292	2.07E-03	3.53	2.61
Vsnl1	17279980	-0.91	-9.74	0.059	1.71E-05	7.47	6.57
Adig	17378898	-0.89	-6.59	0.161	2.37E-04	4.05	3.16
Cpa3	17404337	-0.88	-7.69	0.106	8.57E-05	8.19	7.31
Sprr2b	17399864	-0.86	-5.69	0.207	5.97E-04	5.62	4.76
Apoc1	17487374	-0.85	-6.86	0.161	1.82E-04	6.22	5.37
Gm25683	17482126	-0.82	-5.38	0.231	8.47E-04	6.26	5.43
Ly6d	17318020	-0.82	-4.57	0.295	2.21E-03	5.83	5.01
Pparg	17461942	-0.79	-5.60	0.213	6.64E-04	6.59	5.79
Sik1	17343263	-0.79	-5.13	0.253	1.12E-03	7.57	6.78
Cma1	17306937	-0.78	-5.50	0.228	7.41E-04	5.12	4.34
Apod	17329759	-0.77	-6.10	0.189	3.86E-04	6.69	5.92
Wfdc17	17254289	-0.75	-5.07	0.254	1.20E-03	7.98	7.22
Clec7a	17471541	-0.74	-4.63	0.292	2.05E-03	6.81	6.07
B3galt2	17218006	-0.73	-4.72	0.285	1.84E-03	5.56	4.83
Cdk3-ps	17258555	-0.72	-4.67	0.290	1.95E-03	3.84	3.12
Acaca	17254395	-0.70	-4.62	0.292	2.07E-03	6.51	5.81
Snora33	17239751	-0.69	-4.74	0.283	1.79E-03	6.09	5.40
H2-Aa	17343813	-0.69	-5.18	0.250	1.07E-03	7.56	6.87
DQ267102	17278777	-0.68	-6.55	0.162	2.45E-04	6.45	5.77
Gm23105	17237180	-0.68	-4.72	0.285	1.83E-03	8.91	8.23
Errfi1	17421972	-0.67	-9.27	0.059	2.40E-05	7.25	6.58
Gm23442	17344287	-0.67	-4.52	0.299	2.35E-03	10.22	9.55
Ccl11	17254053	-0.67	-6.63	0.161	2.27E-04	5.94	5.27
Gpd1	17314888	-0.67	-5.38	0.231	8.43E-04	6.53	5.86
Agpat2	17383104	-0.66	-5.94	0.190	4.60E-04	7.35	6.70
Btg2	17227089	-0.63	-4.93	0.269	1.42E-03	7.06	6.43
Epyc	17236821	-0.63	-4.81	0.278	1.65E-03	7.30	6.67
Gstz1	17277592	-0.60	-4.68	0.290	1.91E-03	7.38	6.78
Ranbp1	17328895	-0.60	-5.30	0.239	9.25E-04	6.48	5.89
AF357355	17278775	-0.60	-5.18	0.250	1.07E-03	8.66	8.06
Gm23975	17423505	-0.59	-5.47	0.231	7.67E-04	5.21	4.62
Acp5	17524930	-0.58	-4.77	0.278	1.71E-03	5.92	5.34
Pof1b	17544220	-0.58	-5.82	0.197	5.19E-04	4.29	3.71
Gm24044	17523186	-0.56	-5.90	0.191	4.76E-04	6.69	6.13

Snord14e	17516383	-0.56	-5.01	0.264	1.29E-03	9.12	8.56
Cxcl14	17293006	-0.55	-4.63	0.292	2.05E-03	6.04	5.49
Trf	17530243	-0.54	-4.81	0.278	1.63E-03	9.08	8.54
Gm26448	17523188	-0.52	-5.66	0.207	6.20E-04	7.14	6.61
Folr2	17493949	-0.52	-5.17	0.250	1.08E-03	9.45	8.93
Slc1a5	17474143	-0.51	-5.74	0.204	5.68E-04	7.77	7.26
Mme	17397990	-0.49	-4.58	0.295	2.18E-03	6.92	6.42
Krt80	17321823	-0.47	-5.56	0.215	6.90E-04	5.48	5.01
Adm	17481770	-0.46	-4.52	0.299	2.35E-03	7.63	7.17
Ctss	17400375	-0.46	-4.83	0.278	1.61E-03	8.64	8.18
Ngfrap1	17537895	-0.44	-6.63	0.161	2.28E-04	8.14	7.71
Gm24455	17514824	-0.43	-4.62	0.292	2.07E-03	6.53	6.11
Coq10b	17212874	-0.41	-4.67	0.290	1.95E-03	7.91	7.49
Gm23127	17288714	-0.41	-4.68	0.290	1.93E-03	8.42	8.01
Cfb	17344064	-0.41	-5.37	0.231	8.50E-04	5.60	5.19
Mgst1	17463909	-0.40	-4.62	0.292	2.07E-03	7.02	6.62
Gm10134	17368683	-0.38	-4.54	0.297	2.28E-03	4.54	4.16
Ifi27	17278200	-0.38	-5.48	0.230	7.53E-04	7.83	7.45
Snord95	17248894	-0.37	-4.62	0.292	2.07E-03	8.44	8.07

Total RNA was isolated from atria of postnatal day 4 mice exposed to room air or hyperoxia between PND0-4. The RNA was hybridized to the mouse genome 430 2.0 array from Affymetrix. The mean average signal intensities for each probe and the relative fold change of hyperoxia to room air were determined. The table lists those genes whose expression was significantly decreased by neonatal hyperoxia as defined by a p-value < 0.05 and a false discovery rate of < 0.3. Genes discussed or studied in the paper are highlighted in bold.

TABLE 5.
List of primers used for qRT-PCR

Genes	Primers	GenBank Accession Number	Product Size (bp)
mSLC25a1	F: 5’-AGTGGTGAAGCTGCTCAATAA-3’<br>R: 5’-GCACTGGAATCATCAGAGTAGG-3’	NM153150.2	**115**
mACLY	F: 5’-CGGGAGGAAGCTGATGAATATG-3’<br>R; 5’-GTCAAGGTAGTGCCCAATGAA-3’	NM001199296.1	**91**
mACACA	F: 5’-ACATTCCGAGCAAGGGATAAG-3’<br>R: 5’-GGGATGGCAGTAAGGTCAAA-3’	NM133360.2	96
mACACB	F: 5’-GTCCTGCCCACTTTCTTCTATC-3’<br>R: 5’-GTTTAGCTCGTAGGCGATGTAG-3’	NM133904.2	95
mFASN	F: 5’-AGACCCGAACTCCAAGTTATTC-3’<br>R: 5’-GCAGCTCCTTGTATACTTCTCC-3’	NM007988	101
mSCD1	F: 5’-GGCAGTTCTGAGGTGATTAGAG-3’<br>R: 5’-GTCTCTGGGAAGAGCAATGTAG-3’	NM009127.4	138
mELOVL6	F: 5’-CGCTGAGTGCAACTCTTATCT-3’<br>R: 5’-CCACTAATCTCTGCCCAATCTC-3’	NM130450.2	126
mELOVL4	F: 5’-GACCTGGACCATTGCAGATAA-3’<br>R: 5’-GCCACACGAACAGGAGATAG-3’	NM148941.2	101
mGAPDH	F: 5’-AGGTTGTCTCCTGCGACTTCA-3’<br>R: 5’-CCAGGAAATGAGCTTGACAAAGTT-3’	NM001289726.1	101
mPOL2RA	F: 5’-GCCTCGACTTAAGGAGCTTATC-3’<br>R: 5’-CTCGTGCAGATTGACCTAACA-3’	NM001291068.1	86
mTHRSP	F: 5’-CACCTCTGGGATGTCGTTTAG-3’<br>R: 5’-GGCTTTGGATTCCGTGTTTG-3’	NM009381.3	118
*hSREBF1**	F: 5-’CCCACAACGCCATTGAGAA-3’<br>R: 5’-CAGATTTATTCAGCTTTGCCTCAG-3’	NM001005291.3	100
hKi67	F: 5’-AGACGGCAGTGTATTAG-3’<br>R: 5’-GGCTCTGTCTCAGTATC-3’	NM001145966.2	104
hFASN	F: 5’-TACGACTACGGCCCTCATTT-3’<br>R: 5’-CCATGAAGCTCACCCAGTTATC-3’	NM004104.5	97
hSCD1	F: 5’-ACAACTACCACCACTCCTTTC-3’<br>R: 5’-GGAGACTTTCTTCCGGTCATAG-3’	NM005063.5	128
hGAPDH	F: 5’-CTACATGGCAACTGTGAGGAG-3’<br>R: 5’-CAAGAGCACAAGAGGAAGAGAG-3’	NM001357943.2	102
hPOLR2A	F: 5’-CACCATCAAGAGAGTCCAGTTC-3’<br>R: 5’-CTCAGTCGTCTCTGGGTATTTG-3’	NM000937.5	95

F: forward primer sequence (5’ to 3’); R: reverse primer sequence (5’ to 3’); m = mouse and h = human specific gene names. * Primer recognizes both human and mouse *Srebf1* mRNA.
